# An Umbrella Converse for Data Exchange: Applied to Caching, Computing, and Shuffling †

**DOI:** 10.3390/e23080985

**Published:** 2021-07-30

**Authors:** Prasad Krishnan, Lakshmi Natarajan, V. Lalitha

**Affiliations:** 1 Signal Processing & Communications Research Center, International Institute of Information Technology Hyderabad, Hyderabad 500032, India; lalitha.v@iiit.ac.in; 2 Department of Electrical Engineering, Indian Institute of Technology Hyderabad, Kandi 502205, India; lakshminatarajan@iith.ac.in

**Keywords:** data exchange, coded caching, coded distributed computing, coded data shuffling, converse, index coding

## Abstract

The problem of data exchange between multiple nodes with storage and communication capabilities models several current multi-user communication problems like Coded Caching, Data Shuffling, Coded Computing, etc. The goal in such problems is to design communication schemes which accomplish the desired data exchange between the nodes with the optimal (minimum) amount of communication load. In this work, we present a converse to such a general data exchange problem. The expression of the converse depends only on the number of bits to be moved between different subsets of nodes, and does not assume anything further specific about the parameters in the problem. Specific problem formulations, such as those in Coded Caching, Coded Data Shuffling, and Coded Distributed Computing, can be seen as instances of this generic data exchange problem. Applying our generic converse, we can efficiently recover known important converses in these formulations. Further, for a generic coded caching problem with heterogeneous cache sizes at the clients with or without a central server, we obtain a new general converse, which subsumes some existing results. Finally we relate a “centralized” version of our bound to the known generalized independence number bound in index coding and discuss our bound’s tightness in this context.

## 1. Introduction and Main Result

Consider a system of *K nodes*, denoted by [K]≜{1,⋯,K}, each of which have (not necessarily uniform) storage. The nodes can communicate with each other through a noiseless bus link, in which transmissions of any node is received by all others. Each node possesses a collection of data symbols (represented in bits) in its local storage and demands another set of symbols present in other nodes. We formalize this as a *data exchange problem*.

**Definition** **1.** *A data exchange problem on a set of K nodes involving a collection B of information bits is given by the following:*

- *a collection $\{C_{i}:i\in \left[K\right]\},$ where $C_{i}\subset B$ denotes the subset of data present in node i,*
- *a collection $\left\{D_{i}:i\in \left[K\right]\right\}$ where $D_{i}\subset \cup_{j\neq i}C_{j}\setminus C_{i}$ denotes the set of bits demanded by node i.*

The above data exchange problem models a number of cache-enabled multi-receiver communication problems studied recently in the coding theory community, including Coded Caching [1], Coded Distributed Computing [2,3], Coded Data Shuffling [4,5,6], and Coded Data Rebalancing [7]. In [8], a special case of our general problem here was considered in the name of *cooperative data exchange*, where the goal was to reach a state in which all nodes have all the data in the system.

A solution to a given data exchange problem involves communication between the nodes. Each node *i* encodes the symbols in $C_{i}$ into a codeword of length $l_{i}$ and sends it to all other nodes. The respective demanded symbols at any node is then to be decoded using the received transmissions from all the other nodes and the node’s own content.

Formally, a *communication scheme* for the given data exchange problem consists of a set of encoding functions $\Phi ≜\{\phi_{i}:i\in \left[K\right]\}$ and decoding functions $\Psi ≜\{\psi_{i}:i\in \left[K\right]\}$, defined as follows.

$$\begin{array}{cc} \phi_{i}: & {\left\{0,1\right\}}^{|C_{i}|}\to {\left\{0,1\right\}}^{l_{i}},\left(\mathrm{for}\;\mathrm{some}\;\mathrm{non-negative}\;\mathrm{integer}\;l_{i}\right)\\ \psi_{i}: & {\left\{0,1\right\}}^{|C_{i}|}\times {\left\{0,1\right\}}^{\sum_{j\neq i}l_{j}}\to {\left\{0,1\right\}}^{|D_{i}|}, \end{array}$$

such that

$$\begin{array}{c} \psi_{i}\left(C_{i},\left\{\phi_{j}\left(C_{j}\right):j\neq i\right\}\right)=D_{i}. \end{array}$$

The *communication load* of the above scheme is defined as the total number of bits communicated, i.e.,

$$L\left(\Phi ,\Psi \right)≜\sum_{i\in [K]}l_{i}.$$

The optimal communication load is then denoted by

$$L^{*}≜\min_{\Phi ,\Psi }L\left(\Phi ,\Psi \right).$$

The central result in this work is Theorem 1 in Section 1.1, which is a lower bound on the optimal communication load $L^{*}$. Using this lower bound, we recover several important converse results of cache-enabled communication problems studied in the literature, including Coded Caching (Section 2), Data Shuffling (Section 3), and Distributed Computing (Section 4). In each of these sections, we briefly review each setting and then apply Theorem 1 to recover the respective converses. As a result, the proofs of these existing converses are also made simpler than what is already available in the literature for the respective settings. The generic structure of the converse proofs obtained using our data exchange bound is presented in Section 1.2. This structure includes three steps, which we also highlight at the appropriate junctures within the proofs themselves. The close relationship between these problems is quite widely known. This work gives a further formal grounding to this connection, by abstracting the common structure of these converses into a general form, which can potentially be applied to other new data exchange problems as well.

Apart from recovering existing results, more importantly we also use our data exchange lower bound to obtain *new* tight converse results for some settings, while improving tightness results of some known bounds. Specifically, we present a new converse for a generic coded caching setting with multi-level cache sizes. Using this, we are able to close the gap to optimality for some known special cases of this generic setting (Section 2.1). In Section 5, we show the relationship between a “centralized” version of our data exchange lower bound and an existing bound for index coding known as the α-bound or the generalized independence number bound [9]. In general, we find that our bound is weaker than the α-bound. However, for unicast index coding problems, we identify the precise conditions under which our data exchange bound is equal to the α-bound. In Section 6, we discuss the application of our data exchange lower bound to more generalized index coding settings, specifically distributed index coding [10,11] and embedded index coding [12].

*Notation:* For positive integer *a*, let [a]≜{1,⋯,a}. For a set *S*, we denote by S∖k the set of items in *S* except for the item *k*, and represent the union S∪{k} as S∪k. The binomial coefficient is denoted by nk, which is zero if k>n. The set of all *t*-sized subsets of a set *A* is denoted by At.

### 1.1. A Converse for the Data Exchange Problem

In this subsection, we will obtain a lower bound on the optimal communication load of the general data exchange problem defined in Section 1. This is the central result of this work. The predecessor to the proof technique of our data exchange lower bound is in [3], which first presented an induction based approach for the converse of the coded distributed computing setting. Our proof uses a similar induction technique.

Given a data exchange problem and for P,Q⊂[K] such that P≠∅, let $a_{P}^{Q}$ denote the number of bits which are stored in every node in the subset of nodes *Q* and stored in no other node, and demanded by every node in the subset *P* and demanded by no other node, i.e.,

(1) $$\begin{array}{c} a_{P}^{Q}≜\left|\left(\cap_{i\in P}D_{i}\right)\cap \left(\cap_{j\in Q}C_{j}\right)\setminus \left(\cup_{j\notin Q}C_{j})\cup \left(\cup_{i\notin P}D_{i}\right)\right)\right|. \end{array}$$

Note that, by definition, $a_{P}^{Q}=0$ under the following conditions.

- If P∩Q≠∅, as the bits demanded by any node are absent in the same node.
- If Q=∅, by Definition 1.

Theorem 1 gives a lower bound on the optimal communication load of a given data exchange problem. The proof of the theorem is relegated to Appendix A. The idea of the proof is as follows. If we consider only two nodes in the system, say [K]={1,2}, then each of the 2 nodes has to transmit whatever bits it has which are demanded by the other node, i.e., $L^{*}\geq a_{\left\{2\right\}}^{\left\{1\right\}}+a_{\left\{1\right\}}^{\left\{2\right\}}$. The proof of the theorem uses this as a base case and employs an induction technique to obtain a sequence of cut-set bounds leading to the final expression.

**Theorem** **1.** $$L^{*}\geq \sum_{P\subset [K]}\sum_{Q\subset [K]\setminus P}\frac{\left|P\right|}{|P|+|Q|-1}a_{P}^{Q}.$$

Theorem 1, along with the observation that $a_{\emptyset }^{Q}=0=a_{P}^{\emptyset }$ gives us the following corollary, which is a restatement of Theorem 1.

**Corollary** **1.** *Let*

$$n\left(p,q\right)≜\sum_{\begin{array}{c} P,Q\subset [K]:\\ |P|=p,|Q|=q,P\cap Q=\emptyset \end{array}}a_{P}^{Q}$$

*denote the total number of bits present exactly in q nodes and demanded exactly by p (other) nodes. Then,*

(2) $$\begin{array}{c} L^{*}\geq \sum_{p=1}^{K-1}\sum_{q=1}^{K-p}\frac{p}{p+q-1}n\left(p,q\right). \end{array}$$

**Remark** **1.** *In [13], the authors presented an essentially identical bound (Lemma 1, [13]) as Corollary 1 in the setting of coded distributed computing. The proof given in [13] for this lemma also generalizes the arguments presented in [3], as does this work. Our present work considers a general data exchange problem and derives the lower bound in Theorem 1 for the communication load in such a setting. We had derived this lower bound independently in the conference version of this paper [14], and only recently came to know about the bound in [13]. In subsequent sections, we show how to use this bound to recover converses for various multi-terminal communication problems considered in the literature in recent years, and also obtain new converses for some settings. We also discuss, in Section 5, the looseness of Theorem 1 by considering a centralized version of the data exchange problem and comparing our bound with the generalized independence number bound in index coding. In Section 6, we discuss the application of our data exchange bound to more generalized index coding settings. These are the novel features of our present work, compared to the bound in Lemma 1 of [13].*

### 1.2. A Generic Outline of the Converse Proofs Presented in This Paper

In this work, we derive converse bounds for various settings in coded caching, coded distributed computing, and coded data shuffling using the bound in Theorem 1. Some of these converse bounds are already available in the literature, while others are novel. Each setting enjoins some constraints on the size of the demands and the size of the pre-stored content at each node. The bound in Theorem 1 applies for the setting in which the nodes have some predetermined local storage and some specific demanded bits. However, the settings of coded caching, coded distributed computing, and coded data shuffling permit the design of the initial storage so that the communication load is minimized. Further, the optimal communication load as defined in the literature for some of these settings involves maximization over all possible demand configurations, keeping only the size of the demands fixed. Keeping with these specifics, our bound in Theorem 1 must be tuned for each setting to obtain the respective converse, as captured by the three following steps which describe the generic structure behind our converse proofs.

1. **Applying Theorem 1** to the present setting, we obtain a lower bound expression on the communication load, assuming an arbitrary choice of demands across the nodes and some arbitrary but fixed storage across the nodes.
2. **“Symmetrization” step:** In this step, the lower bound expression obtained in the previous step is averaged over some carefully chosen configurations of demanded bits at the nodes. This step helps to remove the dependency of the lower bound on the specific choice of demands.
3. **Refine the averaged bound** by imposing the constraints on the size of the initial storage at the nodes, and using convexity of terms inside the averaged bound to obtain the final expression of the bound. This step helps to remove the dependency of the converse on the specific initial storage configuration at the nodes.

These three steps enable us to give simpler proofs to those in the literature for known converses, and also obtain novel converses for some variants of the same problems. Further, it also illustrates the generic nature of the data exchange bound of Theorem 1. In the converse proofs that are to follow in this paper, we will highlight these steps at the appropriate junctures.

## 2. Coded Caching

In this section, we apply Theorem 1 to recover the lower bound obtained in [15] for the problem of coded caching introduced in [1]. Further, using Theorem 1, we prove in Section 2.1 a new converse for a generic coded caching problem under multiple cache size settings. This provides new converses for some existing settings in literature, and also tightens bounds in some others. In Section 2.2, we recover a converse for coded caching with multiple file requests. In Section 2.3, we recover the converse for coded caching with decentralized cache placement.

**Figure 1:**
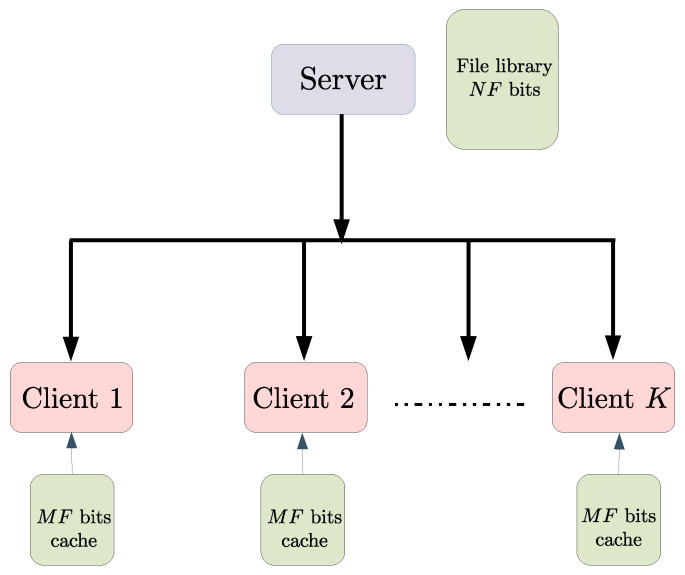
A single server is connected to *K* clients via a broadcast channel. Each user has a cache capable of storing MF of the NF bits in the file-library available at the server.

We now describe the main setting of this section. In the coded caching system introduced in [1], there is one server connected via a noiseless broadcast channel to *K* clients indexed as [K]. The server possesses *N* files, each of size *F* bits, where the files are indexed as $W_{i}:i\in \left[N\right]$. Each client contains local storage, or a *cache*, of size MF bits, for some M≤N. We call this a (K,M,N,F) coded caching system. Figure 1 illustrates this system model.

The coded caching system operates in two phases: in the *caching phase* which occurs during the low-traffic periods, the caches of the clients are populated by the server with some (uncoded) bits of the file library. This is known as *uncoded prefetching*. In this phase, the demands of the clients are not known. We denote the caching function for node *k* as $\zeta_{k},$ and thus the cache content at client *k* at the end of the caching phase is denoted as $Z_{k}≜\zeta_{k}\left(\left\{W_{i}:i\in \left[N\right]\right\}\right)$.

In the *delivery phase* which occurs during the high-traffic periods, each client demands one file from the server, and the server makes transmissions via the broadcast channel to satisfy the client demands. Let the demanded file at client *k* be $W_{d_{k}}$, where $d_{k}\in \left[N\right].$ The server uses an encoding function ϕ to obtain coded transmissions $X=\phi (\left\{W_{d_{k}}:k\in \left[K\right]\right\})$ such that each client k∈[K] can employ a decoding function $\psi_{k}$ to decode its demanded file using the coded transmissions and its cache content, i.e., $\psi_{k}\left(X,Z_{k}\right)=W_{d_{k}}.$

The communication load $L_{c}\left(\left\{\zeta_{k}:k\in \left[K\right]\right\},\phi ,\left\{\psi_{k}:k\in \left[K\right]\right\}\right)$ of the above coded caching scheme is the number of bits transmitted in the delivery phase (i.e., the length of *X*) in the worst case (where “worst case” denotes maximization across all possible demands). The optimal communication load denoted by $L_{c}^{*}$, is then defined as

(3) $$\begin{array}{c} L_{c}^{*}≜\min_{\left\{\zeta_{k}:k\in \left[K\right]\right\},\phi ,\left\{\psi_{k}:k\in \left[K\right]\right\}}L_{c}\left(\left\{\zeta_{k}:k\in \left[K\right]\right\},\phi ,\left\{\psi_{k}:k\in \left[K\right]\right\}\right). \end{array}$$

For this system model, when $\frac{MK}{N}\in Z$,the work in [1] proposed a caching and delivery scheme which achieves a communication load (normalized by the size of the file *F*) given by $K\left(1-\frac{M}{N}\right)\min\left\{\frac{1}{1+\frac{MK}{N}},\frac{N}{K}\right\}$. In [15], it was shown that, for any coded caching scheme with uncoded cache placement, the optimal communication load is lower bounded by $L_{c}^{*}\geq \frac{K(1-\frac{M}{N})F}{1+MK/N}$. Therefore, it was shown that, when K≤N and $\frac{MK}{N}\in Z$, the scheme in [1] is optimal.

In the present section, we give another proof of the lower bound for coded caching derived in [15]. We later discuss the case of arbitrary K,N in Remark 2.

We now proceed with restating the lower bound from [15]. Note that these converses are typically normalized by the file size in literature, however we recall them in their non-normalized form, in order to relate them with our data exchange problem setting.

**Theorem** **2** ([15])**.** Consider a (K,M,N,F) coded caching system with K≤N. The optimal communication load $L_{c}^{*}$ in the delivery phase satisfies

$$L_{c}^{*}\geq \frac{K(1-M/N)}{1+MK/N}F.$$

**Proof** **based** **on** **Theorem** **1.** We assume that the caching scheme and delivery scheme of the coded caching scheme are designed such that the communication load $L_{c}$ is exactly equal to the optimal load $L_{c}^{*}.$ Let the *K* client demands in the delivery phase be represented by a demand vector $d=(d_{1},\cdots ,d_{K})$, where $d_{k}\in \left[N\right]$ denotes the index of the demanded file of the client *k*. We are interested in the worst case demands scenario; this means we can assume that all the demanded files are distinct, i.e., $d_{k}\neq d_{k^{\prime }}$ for all $k\neq k^{\prime }$ to bound $L_{c}^{*}$ from below, without loss of generality.

We observe that a (K,M,N,F) coded caching problem during the delivery phase satisfies Definition 1 of a data exchange problem on K+1 nodes indexed as {0,1,⋯,K}, where we give the index 0 to the server node and include this in the data exchange system. Before proceeding, we remark that the below proof gives a lower bound where all K+1 nodes in the system *may* transmit, whereas in the coded caching system of [1] *only* the server *can* transmit. Thus, any lower bound that we obtain in this proof applies to the setting in [1] also.

Clearly in the equivalent data exchange problem, the node 0 (the server) does not demand anything, but has a copy of all the bits in the entire system. With these observations, we have by definition of $a_{P}^{Q}$ in (1)

(4) aPQ=0,if0∉QorifP∉[K]1,

where the quantities $a_{P}^{Q}$ clearly depend on the demand vector d.

We thus use a new set of variables: for each k∈[K], Q⊂[K], and given demands $d=(d_{1},\cdots ,d_{K})$, let $c_{k}^{Q}\left(d\right)$ denote the number of bits demanded by receiver node *k* that are available only at the nodes Q∪{0}, i.e.,

(5) $$\begin{array}{c} c_{k}^{Q}\left(d\right)≜a_{\left\{k\right\}}^{Q\cup 0}. \end{array}$$

Using these definitions, we proceed following the three steps given in Section 1.2.

**Applying Theorem 1:** By Theorem 1, we have the following lower bound for demand vector d

(6) $$\begin{array}{cc} L_{c}^{*}=L_{c} & \geq \sum_{P\subset [K]\cup \{0\}}\sum_{Q^{\prime }\subset \left[K\right]\cup \left\{0\right\}\setminus P}\frac{\left|P\right|}{\left|P|+\right|Q^{\prime }|-1}a_{P}^{Q^{\prime }}\\ \; & \;\;\;\;=\sum_{k=1}^{K}\sum_{Q\subset [K]\setminus k}\frac{1}{|Q|+1}c_{k}^{Q}\left(d\right), \end{array}$$

where (6) is obtained from (4) and (5).

**“Symmetrizing” (6) over carefully chosen demand vectors:** We now consider the averaging of bounds of type (6) over a chosen collection of *N* demand vectors, given by

(7) $$\begin{array}{cc} D & ≜\left\{\left(j{⊕}_{N}0,j{⊕}_{N}1,\cdots ,j{⊕}_{N}\left(K-1\right)\right):j=0,\cdots ,N-1\right\} \end{array}$$

where $j{⊕}_{N}i≜\left(\left(j+i\right)\;\;\mathrm{mod}\;N\right)+1.$

That is, D contains the demand vectors consisting of consecutive *K* files, starting with each of the *N* files as the demand of the first client.

Averaging (6) through the set of *N* demand vectors in D, the lower bound we obtain is

(8) $$\begin{array}{cc} L_{c}^{*} & \geq \frac{1}{N}\sum_{d\in D}\sum_{k=1}^{K}\sum_{Q\subset [K]\setminus k}\frac{1}{|Q|+1}c_{k}^{Q}\left(d\right). \end{array}$$

Let $b_{n}^{Q}$ denote the number of bits of file *n* stored only in Q∪{0}. Then, in the above sum, $b_{n}^{Q}=c_{k}^{Q}\left(d\right)$ if and only if $d_{k}=n$. This happens precisely once in the collection of *N* demand vectors in D. Thus, we have

$$\begin{array}{cc} L_{c}^{*} & \geq \frac{1}{N}\sum_{k=1}^{K}\sum_{Q\subset [K]\setminus k}\sum_{d\in D}\frac{1}{|Q|+1}c_{k}^{Q}\left(d\right) \end{array}$$

(9) $$\begin{array}{cc} \; & =\frac{1}{N}\sum_{k=1}^{K}\sum_{Q\subset [K]\setminus k}\sum_{n=1}^{N}\frac{1}{|Q|+1}b_{n}^{Q} \end{array}$$

(10) $$\begin{array}{cc} \; & =F\sum_{Q\subset [K]}\sum_{n=1}^{N}\left(\frac{K-|Q|}{|Q|+1}\right)\frac{b_{n}^{Q}}{NF} \end{array}$$

where (10) follows as for a fixed *n* and *Q*, k∈[K]∖Q in (9), and by multiplying and dividing by *F*.

**Refining the bound (10) by using the constraints of the setting:** Now, by definition, $\sum_{n}\sum_{Q\subset [K]}b_{n}^{Q}=NF$, and thus $b_{n}^{Q}/NF:n\in \left[N\right],Q\subset \left[K\right]$, denotes a probability mass function. Furthermore, $\sum_{Q\subset [K]}\left|Q\right|b_{n}^{Q}\leq KMF.$ As (K−x)/(1+x) is a convex decreasing function for x≥0, using Jensen’s inequality, we have $L_{c}^{*}\geq \left(K-x\right)/\left(1+x\right)$, where

$$x=\sum_{n}\sum_{Q\subset [K]}\left|Q\right|\frac{b_{n}^{Q}}{NF}\leq \frac{KMF}{NF}=\frac{KM}{N}.$$

Thus, we get $L_{c}^{*}\geq \frac{K(1-M/N)}{1+MK/N}F,$ which completes the proof. □

**Remark** **2.** In the previous part of this section, we have shown the converse for the worst case communication load $L_{c}^{*}$ for coded caching in the regime of K≤N. We now consider a general coded caching setup with arbitrary K,N values and cache size M. Consider a positive integer $N_{u}\leq \min\left\{N,K\right\}.$ For a fixed caching scheme denoted by $\zeta =\{\zeta_{k}:k\in \left[K\right]\},$ let the minimum communication load for satisfying the clients, maximized across all possible demand vectors with exactly $N_{u}$ distinct files in each of the demand vectors, be denoted as $L_{c}^{*}\left(N_{u},\zeta \right).$

*In the work [16], it was shown that for $t≜\frac{MK}{N},$*

(11) $$\begin{array}{c} L_{c}^{*}\left(N_{u},\zeta \right)\geq g_{{}_{N_{u}}}\left(t\right), \end{array}$$

*where $g_{{}_{N_{u}}}\left(x\right)$ is defined as the lower convex envelope of the points*

P(Nu)=x,Kx+1−K−Nux+1KxF:x∈{0,⋯,K}.

Note that $g_{{}_{N_{u}}}\left(t\right)$ is independent of ζ. For this general setting, the optimal worst case load $L_{c}^{*}$, as defined in (3), satisfies

$$L_{c}^{*}=\min_{\zeta }L_{c}^{*}\left(\min\left\{N,K\right\},\zeta \right).$$

*Thus, from (11), we get*

(12) $$\begin{array}{c} L_{c}^{*}\geq g_{{}_{\min\{N,K\}}}\left(t\right), \end{array}$$

*which is the converse bound on the worst case communication load proved in [16] for this general scenario. In Appendix B, we use our data exchange bound in Theorem 1 to recover (11), which therefore shows (12).*

### 2.1. Server-Based and Server-Free Coded Caching with Heterogeneous Cache Sizes at Clients

So far we have discussed the coded caching scenario where there is a central server containing the entire file library and the client cache sizes are homogeneous, i.e., the same at all clients. We now describe a generalization of the result in Theorem 2 to the case of systems in which the clients have heterogeneous cache sizes, with either a centralized server present or absent. The proof of this is easily obtained from our data exchange bound in Theorem 1. To the best of our knowledge, a converse for this general setting is not known in the literature. Using this converse, we can derive new converses and tighten existing converses for various special cases of this setting, which include widely studied coded caching settings, such as device-to-device coded caching [17].

Consider a coded caching system with *N* files (each of size *F*) with *K* client nodes denoted by a set $K_{T}$. We shall indicate by the value γ the presence (γ=1) or absence (γ=0) of a centralized server in the system containing the file library. For the purpose of utilizing our data exchange bound, we assume that all the nodes in the system are capable of transmissions; thereby, any converse for this scenario is also valid for the usual coded caching scenario in which only the server (if it is present) does transmissions in the delivery phase. The set of clients $K_{T}$ is partitioned into subsets $K_{T_{i}}:i=1,\cdots ,t$ where the nodes in subset $K_{T_{i}}$ can store a fraction $\gamma_{T_{i}}$ of the file library. Let $|K_{T_{i}}|=K_{T_{i}}.$ We now give our converse for this setting. The caching and the delivery scheme, as well as the optimal communication load $L_{c}^{*}$, are defined as in the case of coded caching with homogeneous cache sizes.

**Proposition** **1.** For the above heterogeneous cache sizes setting, assuming K≤N, the optimal communication load $L_{c}^{*}$ for uncoded cache placement is lower bounded as follows.

(13) $$\begin{array}{c} L_{c}^{*}\geq \left(\frac{K-\sum_{i=1}^{t}K_{T_{i}}\gamma_{T_{i}}}{\gamma +\sum_{i=1}^{t}K_{T_{i}}\gamma_{T_{i}}}\right)F. \end{array}$$

Before giving the proof of Proposition 1, we give the following remarks regarding the generality of Proposition 1, the new results which arise by applying Proposition 1 and various results from existing literature that are subsumed or improved by it.

- *Heterogeneous Cache Sizes:* There exists a number of works discussing distinct or heterogenous client cache sizes, for instance, in [18,19]. However, closed form expressions for the lower bound on the load seem to be lacking for such scenarios, to the best of our knowledge. Proposition 1 gives a lower bound for all such settings.
- *Device-to-Device Coded Caching:* Suppose there is no designated server in a coded caching setup, but the client nodes themselves are responsible for exchanging the information to satisfy their demands. This corresponds to the case of Device-to-Device (D2D) coded caching, first explored in [17]. In [17], an achievable scheme was presented for the case when each (client) node has equal cache fraction $\frac{M}{N}$, and this scheme achieves a communication load of $(\frac{N}{M}-1)F$ bits. In the work [20], it was shown that this communication load is optimal (for the regime of K≤N) over all possible “one shot” schemes (where “one shot” refers to those schemes in which each demanded bit is decoded using the transmission only from one server), and further it was shown that the load is within a multiplicative factor of 2 of the optimal communication load under the constraint for uncoded cache placement. We remark that the D2D setting of [17] corresponds to the special case of our current setting, with $\gamma =0,\;t=1,\;K_{T_{1}}=K,\;$ and $\gamma_{T_{1}}=M/N$. By this correspondence, by applying Proposition 1, we see that the load in this case is lower bounded as $\left(\frac{N}{M}-1\right)F,$ thus showing that the achievable scheme in [17] is exactly optimal under uncoded cache placement. The D2D scenario with heterogeneous cache sizes was explored in [21], in which the optimal communication load was characterized as the solution of an optimization problem. However, no closed form expression of the load for such a scenario is mentioned. Clearly, our Proposition 1 gives such a lower bound, when we fix γ=0, for any number of levels *t* of the client-side cache sizes.

Further, the result for coded caching with a server and equal cache sizes at receivers, as in Theorem 2, is clearly obtained as a special case of Proposition 1 with $\gamma =1,\;t=1,\;K_{T_{1}}=K$ and $\gamma_{T_{1}}=\frac{M}{N}$.

We now proceed to prove Proposition 1. The proof is similar to that of Theorem 2.

**Proof** **of** **Proposition** **1.** As in the proof of Theorem 2, we will denote the server node as the node 0 and assume a caching and delivery scheme which achieves the optimal load $L_{c}^{*}$ for worst case client demands.

**Applying Theorem 1**, for our setting, we have

$$\begin{array}{c} L_{c}^{*}\geq \left\{\begin{array}{cc} \sum_{P\subset K_{T}}\sum_{Q\subset K_{T}\setminus P}\frac{\left|P\right|}{|P|+|Q|-1}a_{P}^{Q} & \;\;\;\mathrm{if}\;\gamma =0,\\ \sum_{P\subset K_{T}}\sum_{Q^{\prime }\subset K_{T}\cup 0\setminus P}\frac{\left|P\right|}{\left|P|+\right|Q^{\prime }|-1}a_{P}^{Q^{\prime }} & \;\;\;\mathrm{if}\;\gamma =1. \end{array}\right. \end{array}$$

Note that if γ=1 (i.e., the server is present), then $a_{P}^{Q^{\prime }}=0$ whenever $0\notin Q^{\prime }.$

For a specific demand vector $d=(d_{1},\cdots ,d_{K})$ consisting of distinct demands and for some $Q\subset K_{T}$, we define the quantity $c_{k}^{Q}\left(d\right)$ as follows.

$$\begin{array}{c} c_{k}^{Q}\left(d\right)=\left\{\begin{array}{cc} a_{\left\{k\right\}}^{Q}=\mathrm{Number}\;\mathrm{of}\;\mathrm{bits}\;\mathrm{demanded}\;\mathrm{by}\;k\; & \;\;\;\mathrm{if}\;\gamma =0,\\ \;\;\;\;\;\;\;\;\;\;\;\;\mathrm{available}\;\mathrm{exclusively}\;\mathrm{in}\;Q & \\ a_{\left\{k\right\}}^{Q\cup 0}=\mathrm{Number}\;\mathrm{of}\;\mathrm{bits}\;\mathrm{demanded}\;\mathrm{by}\;k & \;\;\;\mathrm{if}\;\gamma =1.\\ \;\;\;\;\;\;\;\;\;\;\;\;\;\mathrm{available}\;\mathrm{exclusively}\;\mathrm{in}\;Q\cup 0 \end{array}\right. \end{array}$$

**Symmetrization over appropriately chosen demand vectors:** Choosing the same special set of demand vectors D as in (7) and averaging the above lower bound over the demand vectors in D similar to the proof of Theorem 2, we obtain a bound similar to (8):

(14) $$\begin{array}{cc} L_{c}^{*} & \geq \left\{\begin{array}{cc} \frac{1}{N}\sum_{d\in D}\sum_{k\in K_{T}}\sum_{Q\subset K_{T}\setminus k}\frac{c_{k}^{Q}\left(d\right)}{\left|Q\right|} & \;\;\;\mathrm{if}\;\;\gamma =0,\\ \frac{1}{N}\sum_{d\in D}\sum_{k\in K_{T}}\sum_{Q\subset K_{T}\setminus k}\frac{c_{k}^{Q}\left(d\right)}{\left|Q\cup 0\right|} & \;\;\;\mathrm{if}\;\;\gamma =1. \end{array}\right. \end{array}$$

Combining the two expressions in (14), we can write a single equation which holds for γ∈{0,1},

(15) $$\begin{array}{cc} L_{c}^{*} & \geq \frac{1}{N}\sum_{d\in D}\sum_{k\in K_{T}}\sum_{Q\subset K_{T}\setminus k}\frac{c_{k}^{Q}\left(d\right)}{\gamma +|Q|}. \end{array}$$

We now define the term $b_{n}^{Q}$ as follows.

(16) $$\begin{array}{c} b_{n}^{Q}=\left\{\begin{array}{cc} \mathrm{Number}\;\mathrm{of}\;\mathrm{bits}\;\mathrm{of}\;\mathrm{file}\;n\;\mathrm{available}\;\mathrm{exclusively}\;\mathrm{in}\;Q & \;\;\;\mathrm{if}\;\gamma =0,\\ \mathrm{Number}\;\mathrm{of}\;\mathrm{bits}\;\mathrm{of}\;\mathrm{file}\;n\;\mathrm{available}\;\mathrm{exclusively}\;\mathrm{in}\;Q\cup 0 & \;\;\;\mathrm{if}\;\gamma =1. \end{array}\right. \end{array}$$

Using the above definition of $b_{n}^{Q}$ and observing that each demand vector in D has distinct components, Equation (15) can be written as

(17) $$\begin{array}{cc} L_{c}^{*} & \geq \frac{1}{N}\sum_{k\in K_{T}}\sum_{Q\subset K_{T}\setminus k}\sum_{d\in D}\frac{c_{k}^{Q}\left(d\right)}{\gamma +|Q|} \end{array}$$

(18) $$\begin{array}{cc} \; & =\frac{1}{N}\sum_{k\in K_{T}}\sum_{Q\subset K_{T}\setminus k}\sum_{n=1}^{N}\frac{b_{n}^{Q}}{\gamma +|Q|} \end{array}$$

(19) $$\begin{array}{cc} \; & =\frac{1}{N}\sum_{Q\subset K_{T}}\sum_{n=1}^{N}\frac{\left(K-|Q|\right)b_{n}^{Q}}{\gamma +|Q|}. \end{array}$$

**Refining the bound in (19) using setting constraints and convexity:** By the definition of $b_{n}^{Q}$ in (16), we have $\sum_{n}\sum_{Q\subset K_{T}}b_{n}^{Q}=NF$. Further, $\sum_{Q\subset K_{T}}\left|Q\right|b_{n}^{Q}\leq \sum_{i=1}^{t}K_{T_{i}}\gamma_{T_{i}}NF$. Furthermore, for γ≥0, the function $\frac{K-x}{\gamma +x}$ is a convex decreasing function in *x* for x>0. Thus, using Jensen’s inequality, we have $L_{c}^{*}\geq \frac{K-x}{\gamma +x},$ where

$$\begin{array}{c} x=\sum_{n}\sum_{Q\subset K_{T}}\left|Q\right|\frac{b_{n}^{Q}}{NF}\leq \frac{\sum_{i=1}^{t}K_{T_{i}}\gamma_{T_{i}}NF}{NF}=\sum_{i=1}^{t}K_{T_{i}}\gamma_{T_{i}}. \end{array}$$

This completes the proof. □

**Remark** **3.** *Proposition 1 holds when N≥K. This scenario is the most studied case in the literature and is practically more relevant than the case K>N. We now provide lower bounds for the heterogeneous cache sizes setting for general values of K,N, which includes the case K>N. As before, we consider two cases: γ=1 indicates the presence of a centralized server in the system and γ=0 indicates its absence.*

Case 1, γ=1: *For the case where a centralized server is present, i.e., γ=1, we have*

(20) $$\begin{array}{c} L_{c}^{*}\geq g_{\min\{N,K\}}\left(\sum_{i=1}^{t}K_{T_{i}}\gamma_{T_{i}}\right), \end{array}$$

*where the function $g_{\min\{N,K\}}$ is defined in Remark 2. The derivation of this lower bound follows the steps in Appendix B until* (A21)*, where we choose $N_{u}=\min\left\{N,K\right\}$. Without loss of generality, we assume that all caches are fully populated with uncoded bits from the library, thus the total memory occupied by the cached bits $\sum_{Q\subset [K]}\left|Q\right|a^{Q}$ is equal to the sum of all the cache memory available in the system $\sum_{i=1}^{t}K_{T_{i}}\gamma_{T_{i}}NF$. Applying Jensen’s inequality on* (A21) *and using the fact $\sum_{Q\subset [K]}\left|Q\right|\frac{a^{Q}}{NF}=\sum_{i=1}^{t}K_{T_{i}}\gamma_{T_{i}}$, we immediately arrive at the lower bound* (20)*.*

Case 2, γ=0: *In this case, the optimal worst-case communication load can be lower bounded as follows:*

(21) $$\begin{array}{c} L_{c}^{*}\geq \frac{\min\{N,K\}}{K}\left(\frac{K-\sum_{i=1}^{t}K_{T_{i}}\gamma_{T_{i}}}{\sum_{i=1}^{t}K_{T_{i}}\gamma_{T_{i}}}\right)F. \end{array}$$

*The proof of this lower bound follows similar approach as Appendix B and is outlined in Appendix C. Note that when N≥K, both* (20) *and* (21) *become identical to the inequality in Proposition 1.*

### 2.2. Coded Caching with Multiple File Requests

In [22], coded caching with multiple file requests was considered, in which each client requests any Δ files out of the *N* files in the delivery phase. It was shown in [22] (Section V.A) that if the ΔK≤N, then the optimal worst case communication load can be lower bounded as

(22) $$\begin{array}{c} L_{c}^{*}\geq \frac{K\Delta (1-M/N)}{1+MK/N}F. \end{array}$$

The work in [22] also gives an achievable scheme based on the scheme in [1] which meets the above bound. The same lower bound can be derived using Theorem 1 also, by following a similar procedure as that of the proof of Theorem 2.

**Applying Theorem 1,** we give the proof in brief. The demand vector assumed in proof of Theorem 1 becomes a KΔ-length vector in this case, consisting of *K* subvectors, each of length Δ, capturing Δ distinct demands for each client. The proof proceeds as is until (6).

**Symmetrization:** The set D in (7) now contains the KΔ-length vectors of consecutive file indices, cyclically constructed, starting from (1,⋯,KΔ), i.e.,

(23) $$\begin{array}{cc} D & ≜\left\{\left(j{⊕}_{N}0,j{⊕}_{N}1,\cdots ,j{⊕}_{N}\left(K\Delta -1\right)\right):j=0,\cdots ,N-1\right\}. \end{array}$$

Thus, if the demand vector considered is $d\left(j\right)≜\left(j{⊕}_{N}0,j{⊕}_{N}1,\cdots ,j{⊕}_{N}\left(K\Delta -1\right)\right)\in D,$ then the indices of the demanded files at client k∈[K], denoted by $d_{k}\left(j\right),$ is given by

$$\begin{array}{c} d_{k}\left(j\right)≜\left\{j{⊕}_{N}\left(k-1\right)\Delta ,j{⊕}_{N}\left(k-1\right)\Delta +1,\cdots ,j{⊕}_{N}\left(k\Delta -1\right)\right\}. \end{array}$$

The averaged lower bound expression similar to (8) is then obtained as

(24) $$\begin{array}{cc} L_{c}^{*} & \geq \frac{1}{N}\sum_{j=0}^{N-1}\sum_{k=1}^{K}\sum_{Q\subset [K]\setminus k}\frac{1}{|Q|+1}c_{k}^{Q}\left(d\left(j\right)\right). \end{array}$$

In this expression, we have $c_{k}^{Q}\left(d\left(j\right)\right)$ which now indicates the number of bits of Δ distinct and consecutive files indexed by $d_{k}\left(j\right)$ and available exclusively at the nodes in Q∪0 (0 denoting the server).

*Observation:*$c_{k}^{Q}\left(d\left(j\right)\right)=\sum_{n^{\prime }\in d_{k}\left(j\right)}b_{n^{\prime }}^{Q}$ where $b_{n^{\prime }}^{Q}$ denotes the number of bits of file $n^{\prime }$ available exclusively in the nodes Q∪0, as in the proof of Theorem 2.

Now, $n^{\prime }\in d_{k}\left(j\right)$ if and only if the file $n^{\prime }$ is demanded by client *k*. By definition of D, the event $n^{\prime }\in d_{k}\left(j\right)$ happens for precisely Δ values of index *j*. From (24), applying the above observation, we have the following.

(25) $$\begin{array}{cc} L_{c}^{*} & \geq \frac{1}{N}\sum_{k=1}^{K}\sum_{Q\subset [K]\setminus k}\sum_{j=0}^{N-1}\frac{1}{|Q|+1}c_{k}^{Q}\left(d\left(j\right)\right)\\ \; & =\frac{1}{N}\sum_{k=1}^{K}\sum_{Q\subset [K]\setminus k}\frac{1}{|Q|+1}\left(\sum_{j=0}^{N-1}\sum_{n^{\prime }\in d_{k}\left(j\right)}b_{n^{\prime }}^{Q}\right)\\ \; & =\frac{1}{N}\sum_{k=1}^{K}\sum_{Q\subset [K]\setminus k}\sum_{n^{\prime }=1}^{N}\frac{\Delta }{|Q|+1}b_{n^{\prime }}^{Q}. \end{array}$$

**Refining the bound in (25) using the setting constraints:** We use the constraints of the setting and the convexity of the resultant expression to refine (25). This refinement essentially follows similar subsequent steps as in the proof of Theorem 2 following (9), and leads finally to (22).

**Remark** **4.** *The work in [23] considers a coded caching setup in which* Λ *caches (Λ≤K) are shared between the K clients. The special case when* Λ *divides K and each cache is serving exactly $\frac{K}{\Lambda }$ clients is equivalent to the scenario of the multiple file requests in [22] with* Λ *clients, each demanding $\frac{K}{\Lambda }$ files. The above proof then recovers the converse for this setting, which is obtained in [23] (Section III.A in [23]).*

### 2.3. Coded Caching with Decentralized Caching

Theorem 2 and the subsequent results discussed above hold for the *centralized caching* framework, in which the caching phase is designed carefully in a predetermined fashion. In [24], the idea of decentralized placement was introduced, in which the caching phase is not coordinated centrally (this was called “decentralized coded caching” in [24]). In this scenario, each client, independently of others, caches a fraction $\gamma =\frac{M}{N}$ of the bits in each of the *N* files in the file library, chosen uniformly at random. For this scenario, the server (which has the file library) is responsible for the delivery phase. The optimal communication load $L_{c}^{*}$ is defined as the minimum worst case communication load over all possible delivery schemes for a given caching configuration, randomly constructed as given above. For the case of K≤N, the authors of [24] show a scheme which achieves the worst case communication load $L_{c}=KF(1-M/N)\frac{\left(1-{\left(1-M/N\right)}^{K}\right)}{MK/N}$. This was shown to be optimal for large *F* in [16] and also in [25] via a connection to index coding. In the following, we show that the same optimality follows easily via our Theorem 1.

Assume that we have distinct demands at the *K* clients, as in the proof of Theorem 1, given by the demand vector d. We first note that by the law of large numbers, as *F* increases, for the decentralized cache placement, for any k∈[K],Q⊂[K]∖k, we have

$$c_{k}^{Q}\left(d\right)=F{\left(\frac{M}{N}\right)}^{\left|Q\right|}{\left(1-\frac{M}{N}\right)}^{K-|Q|},$$

with probability close to 1, where $c_{k}^{Q}\left(d\right)$ is as defined in (5). This observation enables us to avoid the steps 2 and 3 mentioned in Section 1.2, as the value of $c_{k}^{Q}\left(d\right)$ is independent of the specific random cache placement or the demands chosen (as long as they are distinct). Using this in (6), we get

Lc*≥∑k=1K∑Q⊂[K]∖k1|Q|+1ckQ(d)≥∑k=1K∑Q⊂[K]∖kF|Q|+1MN|Q|1−MNK−|Q|=F∑Q⊂[K]K−|Q||Q|+1MN|Q|1−MNK−|Q|=F∑i=0KKiK−ii+1MNi1−MNK−i=F∑i=0KKi+1MNi1−MNK−i=F∑j=1KKjMNj−11−MNK−j+1=FNM1−MN∑j=1KKjMNj1−MNK−j=NFM1−MN1−1−MNK,

where the last step follows as ∑j=0KKjMNj1−MNK−j=MN+1−MNK=1. Thus, we have given an alternate proof of the optimality of the decentralized scheme in [24].

## 3. Decentralized Coded Data Shuffling

In distributed machine learning systems consisting of a master and multiple worker nodes, data are distributed to the workers by the master in order to perform training of the machine learning model in a distributed manner. In general, this training process takes multiple iterations, with the workers doing some processing (like computing gradients) on their respective training data subsets. In order to ensure that the training data subset at each node are sufficiently representative of the data, and to improve the statistical performance of machine learning algorithms, shuffling of the training data between the worker nodes is implemented after every training iteration. This is known as *data shuffling*.

A coding theoretic approach to data shuffling, which involves the master communicating coded data to the workers was presented in [4]. The setting in [4] was *centralized*, which meant that there is a master node communicating to the servers to perform the data shuffling.

The work in [5] considered the data shuffling problem in which there is no master node, but the worker nodes exchange the training data among themselves, without involving the master node, to create a new desired partition in the next iteration. This was termed as *decentralized data shuffling* in [5]. Note that these notions of “centralized” and “decentralized” in the data shuffling problem are different from those in the coded caching [24], in which these terms were used to define the deterministic and random design of the caching phase, respectively. In this section, we look at the work in [5] and give a new simpler proof of the lower bound on the communication load for decentralized data shuffling.

We first review the setting in [5]. Consider *K* workers in the system, where each worker node is required to process *q* data units at any given time. The total dataset $F_{1}\cup \cdots \cup F_{N}$ consists of N=Kq data units $F_{1},\cdots ,F_{N}$, with a size of *B* bits per data unit. The collection of data units to be processed by worker node *k* at time *t* is denoted as $A_{k,t}$. The collection of data units $A_{1,t},\cdots ,A_{K,t}$ must form a partition of the dataset $F_{1}\cup \cdots \cup F_{N}$ for every time instant *t*, i.e., for any time *t* and any choice of $k,k^{\prime }\in \left[K\right]$ with $k\neq k^{\prime }$ we have

$$\begin{array}{c} A_{k,t}\subset F_{1}\cup \cdots \cup F_{N},\left|A_{k,t}\right|=qB\;\mathrm{and}\;A_{k,t}\cap A_{k^{\prime },t}=\emptyset . \end{array}$$

Each node *k* has a local cache of size MB bits (such that q≤M≤Kq) that can hold *M* data units. Out of these *M* units *q* units are the current “active” data $A_{k,t}$ at any time step which are required to be processed by the node *k*. The contents of the cache of node *k* at time *t* is denoted as $Z_{k,t}$. Therefore, for each choice of k∈[K] and any time *t*, we have

$$\begin{array}{c} |Z_{k,t}|=MB\;\mathrm{and}\;A_{k,t}\subset Z_{k,t}. \end{array}$$

**Figure 2:**
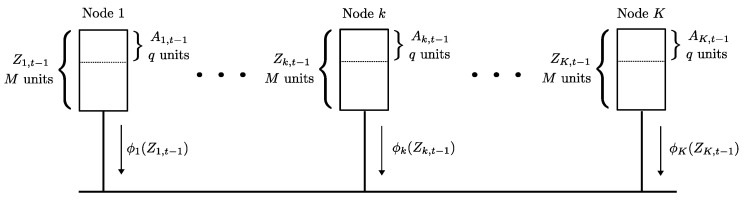
The decentralized data shuffling problem. The contents of the local cache of each node *k* at time t−1 is $Z_{k,t-1}$, out of which a subset $A_{k,t-1}$ is the active data currently processed by the node. The worker nodes must communicate via a broadcast link to shuffle the active data among each other and create a new partition $A_{1,t},\cdots ,A_{K,t}$ of the data units at the next time instance *t*.

At each time instance *t*, a new partition $\left\{A_{k,t}:k\in \left[K\right]\right\}$ is to be made active at the nodes [K], where this new partition is made known to the workers only at time step *t*. Note that the contents of the nodes at time t−1 are $Z_{1,t-1},\cdots ,Z_{K,t-1}$, and the active partition at time t−1 is $A_{1,t-1},\cdots ,A_{K,t-1}$. The worker nodes communicate with each other over a common broadcast link, as shown in Figure 2, to achieve the new partition. The decentralized data shuffling problem is to find a delivery scheme (between workers) to shuffle the collection of active data units $\left\{A_{k,t-1}:k\in \left[K\right]\right\}$ to a new partition $\left\{A_{k,t}:k\in \left[K\right]\right\}$. Each worker *k* computes a function $\phi_{k}\left(Z_{k,t-1}\right)$ of its cache contents and broadcasts it to the other workers. Using these transmissions and the locally available cache content $Z_{k,t-1},$ each node *k* is required to decode $A_{k,t}.$ As in the case of coded caching, one seeks to reduce the worst-case communication load by designing the initial storage and coded transmissions carefully. The communication load of this data shuffling scheme, denoted by $L_{ds}$, is the sum of the number of bits broadcast by all the *K* nodes in the system, i.e., $L_{ds}=\sum_{k\in [K]}\left|\phi_{k}\left(Z_{k,t-1}\right)\right|$. The optimal communication load of data shuffling $L_{ds}^{*}$ (for the worst case data shuffle) is defined as

$$L_{ds}^{*}=\min\max L_{ds}$$

where the maximization is over all possible choices for $A_{k,t-1}:k\in \left[K\right]$ and $A_{k,t}:k\in \left[K\right]$, and the minimization is over all possible choices for the cache placement $\left\{Z_{k,t-1}:k\in \left[K\right]\right\}$ and the delivery scheme $\left\{\phi_{k}:k\in \left[K\right]\right\}$.

For the above setting, the following bound on the communication load $L_{d}^{*}$ was shown in [5].

(26) $$\begin{array}{c} L_{ds}^{*}\geq \frac{Kq}{K-1}.\frac{K-M/q}{M/q}B. \end{array}$$

The above bound was shown to be optimal for some special cases of the parameters, and order-optimal otherwise.

### Proof of the Decentralized Data Shuffling Converse

We now recover the bound (26) by a simple proof using our generic lower bound in Theorem 1. We assume that the cache placement and delivery scheme of the data shuffling scheme are designed such that the communication load of the data shuffling scheme is exactly equal to $L_{ds}^{*}.$ We proceed as per the three steps in Section 1.2.

**Applying Theorem 1:** For k∈[K] and Q⊂[K], let $A_{k,t}^{Q}$ denote the subset of bits of $A_{k,t}$ available exactly at the nodes in *Q* and not anywhere else. Note that $|A_{k,t}^{Q}|=0$ if Q=∅, as each bit is necessarily present in at least one of the *K* nodes.

As per our bound in Theorem 1, we have

$$\begin{array}{c} L_{ds}^{*}=L_{ds}\geq \sum_{k\in [K]}\sum_{Q\subset [K]\setminus k}\frac{|A_{k,t}^{Q}|}{\left|Q\right|}. \end{array}$$

**Symmetrization by averaging over appropriately chosen set of shuffles:** Let the set of circular permutations of (1,2,⋯,K), apart from the identity permutation, be denoted by Γ. There are K−1 of them clearly. We denote an arbitrary permutation in Γ by γ, and by $\gamma_{k}$ we denote the kth coordinate of γ.

Now, consider the shuffle given by γ∈Γ, i.e., for each *k*, $A_{k,t}=A_{\gamma_{k},t-1}$. For this shuffle, we have by the above equation that

(27) $$\begin{array}{cc} L_{ds}^{*} & \geq \sum_{k\in [K]}\sum_{Q\subset [K]\setminus k}\frac{|A_{\gamma_{k},t-1}^{Q}|}{\left|Q\right|} \end{array}$$

(28) $$\begin{array}{cc} \; & =\sum_{Q\subset [K]}\sum_{k\in [K]\setminus Q}\frac{|A_{\gamma_{k},t-1}^{Q}|}{\left|Q\right|}. \end{array}$$

Now, averaging (28) over all permutations in Γ, we get

(29) $$\begin{array}{cc} L_{ds}^{*} & \geq \frac{1}{K-1}\sum_{\gamma \in \Gamma }\sum_{Q\subset [K]}\sum_{k\in [K]\setminus Q}\frac{|A_{\gamma_{k},t-1}^{Q}|}{\left|Q\right|}, \end{array}$$

(30) $$\begin{array}{cc} \; & =\frac{1}{K-1}\sum_{Q\subset [K]}\sum_{k\in [K]\setminus Q}\sum_{\gamma \in \Gamma }\frac{|A_{\gamma_{k},t-1}^{Q}|}{\left|Q\right|}. \end{array}$$

As we go through all choices of γ∈Γ, we see that $\gamma_{k}$ takes every value except *k*, i.e., $\gamma_{k}$ assumes each value in [K]∖k exactly once. Moreover, $A_{k^{\prime },t-1}^{Q}$ is the collection of bits of $A_{k^{\prime },t-1}$ present only in *Q*. However, the bits $A_{k^{\prime },t-1}$ are already presented in $k^{\prime }$. Hence, $|A_{k^{\prime },t-1}^{Q}|=0$ if $k^{\prime }\notin Q$. Therefore, we have

(31) $$\begin{array}{cc} L_{ds}^{*} & \geq \frac{1}{K-1}\sum_{Q\subset [K]}\sum_{k\in [K]\setminus Q}\sum_{k^{\prime }\in Q}\frac{|A_{k^{\prime },t-1}^{Q}|}{\left|Q\right|}, \end{array}$$

(32) $$\begin{array}{cc} \; & =\frac{1}{K-1}\sum_{Q\subset [K]}\sum_{k^{\prime }\in Q}\frac{|A_{k^{\prime },t-1}^{Q}|(K-\left|Q|\right)}{\left|Q\right|} \end{array}$$

(33) $$\begin{array}{cc} \; & =\frac{1}{K-1}\sum_{k^{\prime }\in \left[K\right]}\sum_{Q\subset \left[K\right]:k^{\prime }\in Q}\frac{|A_{k^{\prime },t-1}^{Q}|(K-\left|Q|\right)}{\left|Q\right|}. \end{array}$$

**Refining the bound using setting constraints and convexity:** Now, we have the following observations as $A_{k^{\prime },t-1}^{Q}:k^{\prime }\in \left[K\right],\left\{Q^{\prime }\subset \left[K\right]:k^{\prime }\in Q\right\}$ form a partition of all the NB bits.

$$\begin{array}{cc} \sum_{Q\subset [K]}\sum_{k^{\prime }\in Q}\left|A_{k^{\prime },t-1}^{Q}\right| & =NB=KqB\\ \sum_{Q\subset [K]}\sum_{k^{\prime }\in Q}|A_{k^{\prime },t-1}^{Q}\left||Q\right| & \leq KMB. \end{array}$$

Utilizing the above, and the fact that $\frac{K-|Q|}{\left|Q\right|}$ is a convex decreasing function in |Q| (for |Q|≥0), we have

(34) $$\begin{array}{cc} L_{ds}^{*} & \geq \frac{KqB}{K-1}.\frac{\left(K-\sum_{Q\subset [K]}\sum_{k^{\prime }\in Q}\frac{|A_{k^{\prime },t-1}^{Q}|}{NB}|Q|\right)}{\sum_{Q\subset [K]}\sum_{k^{\prime }\in Q}\frac{|A_{k^{\prime },t-1}^{Q}|}{NB}\left|Q\right|} \end{array}$$

(35) $$\begin{array}{cc} \; & \geq \frac{KqB}{K-1}.\frac{K-KM/N}{KM/N} \end{array}$$

(36) $$\begin{array}{cc} \; & =\frac{KqB}{K-1}.\frac{K-M/q}{M/q} \end{array}$$

Thus, we have recovered (26).

**Remark** **5.** *We have considered the decentralized version of the coded data shuffling problem in this subsection. The centralized version of the data shuffling problem was introduced in [4] and its information theoretic limits were studied elaborately in [6]. Our data exchange bound, when applied to the setting in [6], results in a looser converse result than that in [6]. The reasons for this is explored in Section 5 using the connection between our data exchange bound and the bound for index coding known in literature.*

## 4. Coded Distributed Computing

In a distributed computing setting, there are *N* files on which the distributed computing task has to be performed by *K* nodes. The job at hand is divided into three phases: Map, Shuffle, and Reduce. In the shuffle phase, the nodes that are assigned to perform the distributed computing task exchange data. In [3], the authors proposed coded communication during the shuffle phase to reduce the communication load. We recollect the setting and the main converse result from [3], which we recover using our data exchange bound.

**Figure 3:**
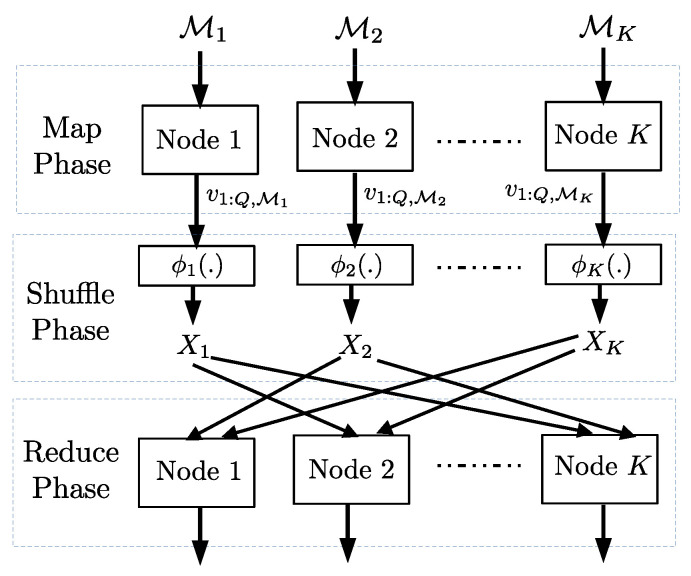
There are *N* files and a subset $M_{i}$ of them are assigned to node *i* in map phase. The output of map phase at each node is $v_{1:Q,M_{i}}$. Each node computes $X_{i}=\phi_{i}\left(v_{1:Q,M_{i}}\right)$ which it broadcasts to other nodes. The nodes compute the reduce outputs based on their own map outputs and the broadcasts which they receive.

A subset $M_{i}$ of *N* files is assigned to ith node and the ith node computes the map functions on this subset in the map phase (see Figure 3). We assume that the total number of map functions computed at the *K* nodes is rN, where *r* is referred to as the *computation load*. In the reduce phase, a total of *W* reduce functions is to be computed across the *K* nodes corresponding to the *N* files. Each node is assigned the same number of functions. Obtaining the output of the reduce functions at all the nodes will complete the distributed computing task. In this work, as in [3], we consider two scenarios: in the first one, each reduce function is computed exactly at one node and in the second, each reduce function is computed at *s* nodes, where s≥2.

Each map function output (also referred to as intermediate output) corresponds to a particular file and a particular reduce function. For each file and each reduce function, an intermediate output of *T* bits is obtained. To compute an assigned reduce function, each node requires the intermediate outputs of *all* the files corresponding to the assigned reduce function. This means each node is missing the intermediate outputs (corresponding to the assigned reduce functions) of those files that are not assigned to it in the map phase.

The intermediate outputs of each file assigned to node *i* corresponding to all the reduce functions are available at node *i* at the end of the map phase and denoted by $v_{1:Q,M_{i}}$. These intermediate outputs at the end of the map phase are encoded as follows: $X_{i}=\phi_{i}\left(v_{1:Q,M_{i}}\right)$ and broadcasted to the remaining nodes, in the shuffle phase (in order to deliver the missing intermediate outputs at the nodes). Let $L_{dc}^{*}$ be the total number of bits broadcasted by the *K* nodes in the shuffle phase, minimized over all possible map function assignments, reduce function assignments, and shuffling schemes, with a computation load *r*. We refer to $L_{dc}^{*}$ as the *minimum communication load*.

To obtain similar expressions for the communication load as in [3], we normalize the communication load by the total number of intermediate output bits (=WNT). We consider the first scenario now, where each reduce function is computed exactly at one node.

**Theorem** **3** ([3])**.** The minimum communication load $L_{dc}^{*}$ incurred by a distributed computing system of K nodes for a given computation load r, where every reduce function is computed at exactly one node and each node computes $\frac{W}{K}$ reduce functions, is bounded as

(37) $$\frac{L_{dc}^{*}}{WNT}\geq \frac{1}{r}\left(1-\frac{r}{K}\right).$$

**Proof.**  We resort to two of the three steps of Section 1.2 to complete this proof. The symmetrization step, which involves averaging over demand configurations, is not applicable in the present setting because the definition of $L_{dc}^{*}$ involves minimization over the reduce function assignment as well.

**Applying Theorem 1:** Let $M=(M_{1},\ldots ,M_{K})$ denote a given map function assignment to the nodes, where $M_{i}\subset \left[N\right]$. Let $L_{M}$ denote the communication load associated with the map function assignment M. We will prove that

$$\frac{L_{M}}{WNT}\geq \sum_{j=1}^{K}\frac{{\tilde{a}}_{M}^{j}}{N}\frac{K-j}{Kj},$$

where ${\tilde{a}}_{M}^{j}$ denotes the number of files which are mapped at exactly *j* nodes in [K]. It is easy to see that $\sum_{j=1}^{K}{\tilde{a}}_{M}^{j}=N$ and $\sum_{j=1}^{K}j{\tilde{a}}_{M}^{j}=rN$. We will apply Theorem 1 to this setting. Recall that each reduce function is computed exactly at one node in our present setup. To apply Theorem 1, we need to ascertain the quantities $a_{P}^{Q}$ for P,Q being disjoint subsets of [K]. To do this, we first denote by ${\tilde{a}}^{Q}$ the number of files whose intermediate outputs are demanded by some node *k* and available exclusively in the nodes of *Q*. Note that ${\tilde{a}}^{Q}$ is the same for any k∈[K]∖Q, as each node demands intermediate outputs of *all* the files that are not mapped at the node itself.

As the number of reduce functions assigned to node *k* is $\frac{W}{K}$ (as each reduce function is computed at exactly one node) and each intermediate output is *T* bits, the number of intermediate output bits which are demanded by any node *k* and available exclusively in the nodes of *Q* are $\frac{WT}{K}{\tilde{a}}^{Q}$. Thus, for any Q⊂[K], the quantities $a_{P}^{Q}$ in Theorem 1 are given as follows.

$$a_{P}^{Q}=\left\{\begin{array}{cc} \frac{WT}{K}{\tilde{a}}^{Q} & \mathrm{if}\;P=\{k\}\;\mathrm{for}\;\mathrm{some}\;k\in [K]\;\mathrm{such}\;\mathrm{that}\;k\notin Q\\ 0 & \mathrm{otherwise} \end{array}\right.$$

Further note that $\sum_{Q\subset [K]:|Q|=j}{\tilde{a}}^{Q}={\tilde{a}}_{M}^{j}$ by definition of ${\tilde{a}}_{M}^{j}.$ Using these and applying Theorem 1 with the normalization factor WNT, we have the following inequalities.

$$\begin{array}{cc} \frac{L_{M}}{WNT} & \geq \frac{1}{WNT}\sum_{k=1}^{K}\sum_{Q\subset [K]\setminus \{k\}}\frac{1}{\left|Q\right|}{\tilde{a}}^{Q}\frac{WT}{K}\\ \; & =\frac{1}{KN}\sum_{j=1}^{K}\sum_{Q\subset [K]:|Q|=j}\sum_{k\in [K]\setminus Q}\frac{1}{j}{\tilde{a}}^{Q}\\ \; & =\frac{1}{KN}\sum_{j=1}^{K}\sum_{Q\subset [K]:|Q|=j}\frac{K-j}{j}{\tilde{a}}^{Q}\\ \; & =\frac{1}{KN}\sum_{j=1}^{K}\frac{K-j}{j}\left(\sum_{Q\subset [K]:|Q|=j}{\tilde{a}}^{Q}\right)\\ \; & =\frac{1}{KN}\sum_{j=1}^{K}\frac{K-j}{j}{\tilde{a}}_{M}^{j}. \end{array}$$

**Refining the bound using convexity and setting constraints:** Using definition of $L_{dc}^{*}$, noting that $\frac{K-j}{j}$ is a convex decreasing function of *j* and that $\frac{\sum_{j=1}^{K}{\tilde{a}}_{M}^{j}}{N}=1$, we have that

$$\begin{array}{cc} \frac{L_{dc}^{*}}{WNT} & \geq \frac{1}{K}\frac{K-\sum_{j=1}^{K}\frac{j{\tilde{a}}_{M}^{j}}{N}}{\sum_{j=1}^{K}\frac{j{\tilde{a}}_{M}^{j}}{N}}\\ \; & =\frac{1}{K}\frac{K-r}{r}=\frac{1}{r}\left(1-\frac{r}{K}\right). \end{array}$$

□

Now, we consider the case in which each reduce function has to be computed at *s* nodes. The total number of reduce functions is assumed to be *W*. In addition, the following assumption is made to keep the problem formulation symmetric with respect to reduce functions: every possible *s* sized subset of *K* nodes is assigned WKs reduce functions (we assume Ks divides *W*). As in the previous case, we will denote the communication load for a given map function assignment by $L_{M}\left(s\right)$ and the optimal communication load with computation load *r* by $L_{dc}^{*}\left(s\right)$. We will prove the following result which gives a lower bound on $L_{M}\left(s\right)$.

**Proposition** **2** ([3])**.** *The communication load corresponding to a map function assignment M when each reduce function has to be computed at s nodes is lower bounded as*

(38) LM(s)WNT≥∑j=1Ka˜MjN∑l=max(0,s−j)min(K−j,s)K−jljs−lKsll+j−1.

**Proof.**  As before, we will denote by ${\tilde{a}}^{Q}$ the number of files whose map function outputs are available exclusively in the nodes of *Q*. Furthermore, we will denote the number of intermediate output bits which are demanded exclusively by the nodes in *P* and available exclusively in the nodes of *Q* by $b_{P}^{Q}$. Then, applying Theorem 1, the lower bound on the communication load in terms of $\left\{b_{P}^{Q}\right\}$ is given by

$$\begin{array}{ccc} \frac{L_{M}\left(s\right)}{WNT} & \geq & \frac{1}{WNT}\sum_{P\subset [K]}\sum_{Q\subset [K]\setminus P}\frac{\left|P\right|}{|P|+|Q|-1}b_{P}^{Q}. \end{array}$$

We first interchange the above summation order and consider all sets *Q* with |Q|=j and all sets *P* such that |P|=l. For |Q|=j, we need to count the subsets of size *s*, which form a subset of P∪Q. Thus, for a fixed *j*, we can see that the range of *l* can vary from max(0,s−j) to min(K−j,s). For a given subset *P* of size *l*, the number of *s* sized subsets which are contained within P∪Q and contain *P* are js−l. Therefore, the number of intermediate output bits demanded exclusively by the nodes in *P* and available exclusively in *Q*, $b_{P}^{Q}$, is given by bPQ=a˜QWTjs−lKs. This is because each of the *s*-sized subset has to reduce a˜QWKs functions. Using this relation, the above inequality can be rewritten as follows.

(39) LM(s)WNT≥∑j=1K1N∑Q⊂[K]:|Q|=ja˜Q∑l=max(0,s−j)min(K−j,s)ll+j−1∑P⊂[K]∖Q:|P|=ljs−lKs

(40) =∑j=1Ka˜MjN∑l=max(0,s−j)min(K−j,s)ll+j−1K−jljs−lKs,

where (40) follows as ${\tilde{a}}_{M}^{j}=\sum_{Q\subset [K]:|Q|=j}{\tilde{a}}^{Q}$. This completes the proof. □

The above lemma along with certain convexity arguments resulting from the constraints imposed by the computation load can be used to prove the lower bound on $L_{dc}^{*}\left(s\right)$. The interested reader is referred to the converse proof of Theorem 2 in [3] for the same.

## 5. Relation to Index Coding Lower Bound

We now consider the “centralized” version of the data exchange problem, where one of the nodes has a copy of all the information bits and is the lone transmitter in the system. We will use the index 0 for this server node, and assume that there are *K* other nodes in the system, with index set [K], acting as clients. In terms of Definition 1, this system is composed of K+1 nodes {0}∪[K], the demand $D_{0}$ of the server is empty, while the demands $D_{i}$ and the contents $C_{i}$ of all the clients are subsets of the contents of the server, i.e., $C_{i},D_{i}\subset C_{0}$ for all i∈[K]. Without loss of generality, we assume that only the server performs all the transmissions as any coded bit that can be generated by any of the client nodes can be generated at the server itself. Clearly, this is an index coding problem [26] with *K* clients or receivers, the demand of the ith receiver is $D_{i}$, and its *side information* is $C_{i}$. When applied to this scenario, our main result Theorem 1 therefore provides a lower bound on the index coding communication cost.

The maximum acyclic induced subgraph (MAIS) and its generalization, which is known as the generalized independence number or the α-bound, are well-known lower bounds in index coding [9,26]. In this section, we describe the relation between the α-bound of index coding and the centralized version of Theorem 1. We show that the latter is in general weaker, and identify the scenarios when these two bounds are identical. We then use these observations to explain why Theorem 1 cannot provide a tight lower bound for the centralized data shuffling problem [6].

Let us first apply Theorem 1 to the centralized data exchange problem. As node 0 contains all the information bits and its demand is empty, we have $a_{P}^{Q^{\prime }}=0$ if $0\notin Q^{\prime }$ or 0∈P. Using $Q=Q^{\prime }\setminus \left\{0\right\}$ and defining the variable $c_{P}^{Q}=a_{P}^{Q\cup \{0\}}=a_{P}^{Q^{\prime }}$, we obtain

**Theorem** **4.** *The centralized version of our main result Theorem 1 is*

$$\begin{array}{cc} L^{*} & \geq \sum_{P\subset [K]}\sum_{\begin{array}{c} Q^{\prime }\subset \left\{0\right\}\cup \left[K\right]\\ 0\in Q^{\prime },P\cap Q^{\prime }=\emptyset \end{array}}\frac{\left|P\right|}{\left|P|+\right|Q^{\prime }|-1}a_{P}^{Q^{\prime }}\\ \; & =\sum_{P\subset [K]}\sum_{Q\subset [K]\setminus P}\frac{\left|P\right|}{\left|P|+|Q\right|}c_{P}^{Q}. \end{array}$$

Note that it is possible to have $c_{P}^{Q}=a_{P}^{Q\cup \{0\}}>0$ when Q=∅.

In Section 5.1, we express the generalized independence number α in terms of the parameters $c_{P}^{Q}$, and in Section 5.2, we identify the relation between our lower bound Theorem 4 and the index coding lower bound α.

### 5.1. The Generalized Independence Number Bound

Let $\gamma =(\gamma_{1},\cdots ,\gamma_{K})$ be any permutation of [K], where $\gamma_{i}$ is the ith coordinate of the permutation. Applying similar ideas as in the proof of Theorem 1 to the centralized scenario, we obtain the following lower bound on $L^{*}$. This lower bound considers the nodes in the order $\gamma_{1},\cdots ,\gamma_{K}$, and for each node in this sequence it counts the number of bits that are demanded by this node which are neither demanded by and nor available as side information in any of the earlier nodes.

**Proposition** **3.** *For any permutation γ of [K],*

(41) $$L^{*}\geq \sum_{i=1}^{K}\;\sum_{\begin{array}{c} P\subset \{\gamma_{i},\cdots ,\gamma_{K}\}\\ \gamma_{i}\in P \end{array}}\;\sum_{Q\subset \{\gamma_{i+1},\cdots ,\gamma_{K}\}}c_{P}^{Q}.$$

**Proof.**  See Appendix D. □

A direct consequence of Proposition 3 is

(42) $$L^{*}\geq \max_{\gamma }\sum_{i=1}^{K}\;\sum_{\begin{array}{c} P\subset \{\gamma_{i},\cdots ,\gamma_{K}\}\\ \gamma_{i}\in P \end{array}}\;\sum_{Q\subset \{\gamma_{i+1},\cdots ,\gamma_{K}\}}c_{P}^{Q}$$

where the maximization is over all possible permutations on [K].

We now recall the definition of the generalized independence number [9]. Denote the collection of the $c_{P}^{Q}$ information bits available exclusively at the nodes Q∪{0} and demanded exclusively by the nodes *P* as $\left\{w_{P,m}^{Q}\;:\;m=1,\cdots ,c_{P}^{Q}\right\}$. Therefore, the set of all the information bits present in the system is

$$B=\underset{P\subset [K]}{⋃}\underset{Q\subset [K]\setminus P}{⋃}\left\{w_{P,m}^{Q}\;:\;m=1,\cdots ,c_{P}^{Q}\right\}.$$

Note that each bit is identified by a triple (P,Q,m).

**Definition** **2.** *A subset H of B is a generalized independent set if and only if every subset I⊂H satisfies the following:*

- *there exists a node k∈[K] and an information bit in I such that this information bit is demanded by k (and possibly some other nodes), and none of the other bits in I are available as side information at k.*

*The generalized independence number α is the size of the largest generalized independent set.*

We next show that the lower bound in (42) is in fact equal to the generalized independence number α of this index coding problem.

**Theorem** **5.** *The generalized independence number α satisfies*

(43) $$\alpha =\max_{\gamma }\sum_{i=1}^{K}\;\sum_{\begin{array}{c} P\subset \{\gamma_{i},\cdots ,\gamma_{K}\}\\ \gamma_{i}\in P \end{array}}\;\sum_{Q\subset \{\gamma_{i+1},\cdots ,\gamma_{K}\}}c_{P}^{Q},$$

*where the maximization is over all K! permutations of [K].*

**Proof.**  See Appendix E. □

### 5.2. Relation to the Index Coding Lower Bound

Proposition 3 serves as the platform for comparing Theorem 4 and the α-bound. While α equals the *maximum* value of the bound in Proposition 3 over all permutations on [K], our bound in Theorem 4 equals the *average* value of the lower bound given in Proposition 3 over all permutations on [K]. We will show this relation between Theorem 4 and Proposition 3 now.

Taking the average of the right hand side of (41) with respect to all γ, we obtain

$$\frac{1}{K!}\sum_{\gamma }\;\sum_{i=1}^{K}\sum_{\begin{array}{c} P\subset \{\gamma_{i},\cdots ,\gamma_{K}\}\\ \gamma_{i}\in P \end{array}}\sum_{Q\subset \{\gamma_{i+1},\cdots ,\gamma_{K}\}}c_{P}^{Q}.$$

For each choice of P,Q⊂[K] with P∩Q=∅, we now count the number of times $c_{P}^{Q}$ appears in this sum. For a given γ, the inner summations include the term $c_{P}^{Q}$ if and only if the following holds:

$$\gamma_{i}\in P,\;\mathrm{where}\;i=\min\left\{j\in \left[K\right]\;:\;\gamma_{j}\in P\cup Q\right\},$$

i.e., if we consider the elements $\gamma_{1},\cdots ,\gamma_{K}$ in that order, the first element from P∪Q to be observed in this sequence belongs to *P*. Thus, for a given pair P,Q the probability that a permutation γ chosen uniformly at random includes the term $c_{P}^{Q}$ in the inner summation is |P|/(|P|+|Q|). Therefore, the average of the lower bound in Proposition 3 over all possible γ is

$$\sum_{P\subset [K]}\sum_{\begin{array}{c} Q\subset [K]\\ P\cap Q=\emptyset \end{array}}\frac{\left|P\right|}{\left|P|+|Q\right|}c_{P}^{Q},$$

which is exactly the bound in Theorem 4.

As the bound in Theorem 4 is obtained by averaging over all γ, instead of maximizing over all γ, we conclude that this is in general weaker than the α-bound of index coding. The two bounds are equal if and only if the bound in Proposition 3 has the same value for every permutation γ.

Although weaker in general, we note that the bound of Theorem 4 is easier to use than the α-bound. As demonstrated by (2), in order to use Theorem 4, we only need to know, for each information bit, the number of nodes that contain this bit and the number of nodes that demand this bit. In comparison, this information is insufficient to evaluate the α-bound, which also requires the identities of these nodes.

### 5.3. On the Tightness of Theorem 4

We now consider the class of *unicast* problems, i.e., problems where each bit is demanded by exactly one of the nodes. For this class of problems, we characterize when Theorem 4 yields a tight bound.

**Theorem** **6.** For unicast problems the bound in Theorem 4 equals $L^{*}$ if and only if every S⊂[K] with |S|≥2 satisfies the following, $c_{\left\{k\right\}}^{S\setminus k}=c_{\left\{k^{\prime }\right\}}^{S\setminus k^{\prime }}$ for every $k,k^{\prime }\in S$.

**Proof.**  See Appendix F. When the lower bound of Theorem 4 is tight, the clique-covering based index coding scheme (see in [26,27]) yields the optimal communication cost. □

Our main result in Theorem 1, or equivalently, Theorem 4, does not provide a tight lower bound for centralized data shuffling problem [6], because this problem involves scenarios that do not satisfy the tightness condition of Theorem 6. For instance, consider the simple canonical data shuffling setting, where the system has exactly *K* files, all of equal size *F* bits, and each node stores exactly one of these files, i.e., the entirety of the contents of the kth node $C_{k}$ is the kth file. Here, $|C_{k}|=F$ for all k∈[K], and $C_{i}\cap C_{j}=\emptyset$ for all i≠j. Assume that the shuffling problem is to move the file $C_{k+1}$ to node *k*, i.e., $D_{k}=C_{k+1}$, where we consider the index K+1 to be equal to 1. This is a worst-case demand for data shuffling incurring the largest possible communication cost. For this set of demands, we have $c_{k}^{\left\{k+1\right\}}=F$ for all k∈[K], and $c_{k}^{Q}=0$ for all other choices of k,Q. In particular, $c_{k+1}^{\left\{k\right\}}=0\neq c_{k}^{\left\{k+1\right\}}$. Clearly, the condition in Theorem 6 does not hold for S={k,k+1}. Therefore, our lower bound is strictly less than $L^{*}$ for this data shuffling problem, and therefore is not tight.

## 6. Relationship to Other Index Coding Settings

We now comment on the application of our data exchange bound to a couple of other important index coding settings known in literature, (a) *distributed index coding* studied in [10] (which is equivalent to the *cooperative multi-sender index coding* setting considered in [11]), and (b) *embedded index coding*, presented in [12].

### 6.1. Distributed Index Coding

In [10], the authors consider a generalization of the single-server index coding problem (which we studied in Section 5) called *distributed index coding*. The specific setting in [10] is as follows. There are *n* messages denoted by $x_{j}:j\in \left[n\right],$ where $x_{j}\in {\left\{0,1\right\}}^{t_{j}}$ (for some positive integer $t_{j}$). There is a corresponding set of *n* receivers indexed by [n]. The receiver j∈[n] contains as side-information the subset of messages indexed by $A_{j}\subset \left[n\right]$ (i.e., receiver *j* knows $\left\{x_{i}:i\in A_{j}\right\}$) and demands the message $x_{j}$. There are $2^{n}-1$ servers in the system, indexed by the sets J={J:J⊂[n],J≠∅}. The server *J* contains the messages $\{x_{i}:i\in J\}.$ The servers do not demand any messages and are responsible only for transmissions that satisfy the receivers. The server *J* is connected to the *n* receivers via a broadcast link with capacity $C_{J}$ bits. In order to satisfy the demands, each server *J* sends a message $y_{J}\in {\left\{0,1\right\}}^{s_{J}}$ to all the receivers, where $s_{J}$ is some positive integer.

**Definition** **3** ([10])**.** *The rate-capacity tuple $\left(\left(R_{j}:j\in \left[n\right]\right),\left(C_{J}:J\in J\right)\right)$ is said to be achievable if there exists some positive integer r such that $t_{j}\geq rR_{j},\forall j$ and $s_{J}\leq rC_{J},\forall J,$ and there exists valid encoding functions (encoding the messages of lengths $\left(t_{j}:j\in \left[n\right]\right)$ into codewords of lengths $\left(s_{J}:J\in J\right)$) and decoding functions, such that all receivers can decode their respective demands.*

Slightly abusing Definition 2, for some T⊂[n], we call a set S⊂T of message indices as a generalized independent set of *T*, if for every subset $S^{\prime }\subset S,$ there is some $j\in S^{\prime }$ such that $A_{j}\cap \left(S^{\prime }\setminus j\right)=\emptyset .$

Let $\left(\left(R_{j}:j\in \left[n\right]\right),\left(C_{J}:J\in J\right)\right)$ be an achievable rate-capacity tuple. For any non-empty subset T⊂[n], let $S_{T}$ be a generalized independent set of *T*. In Corollary 2 of [10], it is shown that

(44) $$\begin{array}{c} \sum_{j\in S_{T}}R_{j}\leq \sum_{J:J\cap T\neq \emptyset }C_{J}. \end{array}$$

**Remark** **6.** *The above bound in (44) is given in [10] using the terminology of the side-information graph defining the index coding problem and its acyclic induced subgraphs. However, we have used generalized independent sets to state the same bound. The reader can easily confirm that the acyclic induced subgraph of the side-information graph as defined in [10] is the same as a generalized independent set we have used in this work. Therefore, (44) is the same as the bound in Corollary 2 of [10].*

Let

$$S_{max}=arg\max_{S}\sum_{j\in S}t_{j},$$

where the maximization is over all generalized independent sets *S* of [n].

Then, we have by (44),

(45) $$\begin{array}{c} \sum_{j\in S_{max}}R_{j}\leq \sum_{J\in J}C_{J}. \end{array}$$

In order to relate the bound in (44) with our data exchange bound, we fix $R_{j}=t_{j},\forall j\in \left[n\right]$. This means that we should have r=1 in Definition 3. For these parameters, let $s_{J}^{*}:J\in J$ be a choice of integers $s_{J}:J\in J$ such that the rate-capacity tuple $\left(\left(R_{j}=t_{j}:j\in \left[n\right]\right),\left(s_{J}:J\in J\right)\right)$ is achievable and $\sum_{J\in J}s_{J}$ is minimized. Note that such integers $s_{J}^{*}:J\in J$ will exist as each index coding problem has at least one solution, namely, the trivial solution consisting of uncoded transmissions of $x_{j}:j\in \left[n\right]$.

Then, applying (45), we have

(46) $$\begin{array}{c} \sum_{j\in S_{max}}t_{j}\leq \sum_{J\in J}s_{J}^{*}. \end{array}$$

Note that $\sum_{J\in J}s_{J}^{*}$ is exactly the minimum number of bits to be communicated by the servers for satisfying receiver demands.

For Q⊂[n], define

$$\begin{array}{c} f_{j}^{Q}≜\left\{\begin{array}{cc} 1 & \mathrm{if}\;j\in \left(\cap_{k\in Q}A_{k}\right)\setminus \left(\cup_{k^{\prime }\in \left[n\right]\setminus Q}A_{k^{\prime }}\right),\\ 0 & \mathrm{otherwise}. \end{array}\right. \end{array}$$

By arguments similar to that of the proof of Theorem 5, we can verify that

(47) $$\begin{array}{c} \sum_{j\in S_{max}}t_{j}=\max_{\gamma }\sum_{j=1}^{n}\sum_{Q\subset \{\gamma_{j+1},\cdots ,\gamma_{n}\}}f_{\gamma_{j}}^{Q}t_{\gamma_{j}}, \end{array}$$

where the maximization is over all possible permutations $\gamma =(\gamma_{1},\cdots ,\gamma_{n})$ of (1,⋯,n). We thus have by (46) and (47),

(48) $$\begin{array}{c} \sum_{J\in J}s_{J}^{*}\geq \max_{\gamma }\sum_{j=1}^{n}\sum_{Q\subset \{\gamma_{j+1},\cdots ,\gamma_{n}\}}f_{\gamma_{j}}^{Q}t_{\gamma_{j}}. \end{array}$$

Finally, we apply our data exchange bound in Theorem 1 to the distributed index coding setting. To do this, we first observe that if we replaced all servers by a single “virtual” central server containing all the messages, $x_{j}:j\in \left[n\right],$ then $\sum_{J\in J}s_{J}^{*}$ is the minimum number of bits to be transmitted by this virtual central server to satisfy the receiver demands. Any lower bound on the communication cost for this transformed setting with the virtual server will thus continue to apply for the original distributed setting with messages of length $t_{j}:j\in \left[n\right]$. Now, utilizing the centralized version of Theorem 1 shown in Theorem 4 and by the discussion in Section 5.2, we get

(49) $$\begin{array}{c} \sum_{J\in J}s_{J}^{*}\geq \frac{1}{n!}\sum_{\gamma }\sum_{j=1}^{n}\sum_{Q\subset \{\gamma_{j+1},\cdots ,\gamma_{n}\}}f_{\gamma_{j}}^{Q}t_{\gamma_{j}}. \end{array}$$

Therefore, we see that the generalized independent set based bound in (48) is in general better than (49), as (48) involves a maximization over all permutations γ, while (49) involves the average.

### 6.2. Embedded Index Coding

We now consider the embedded index coding problem, introduced in [12], motivated by device-to-device communications. The embedded index coding setting consists of a set of *m* data blocks (each a binary vector of length *t*) distributed across a set of *n* nodes. Each node stores (as side information) a subset of the data blocks and demands another subset which it already does not have. This setting is different from [26,27] or distributed index coding [10], as there are no dedicated servers by default here. Each node transmits a codeword obtained by encoding its data blocks, and each demanded data block at any node is decoded from the codewords obtained from other nodes and the side information at the node itself. An *embedded index code* consists of a collection of such encoding functions and decoding functions at the nodes, such that all demanded blocks are decoded at the respective nodes. The *communication cost* of embedded index coding is the total number of bits transmitted between the nodes to satisfy the node demands. The work [12] generalizes the notion of *minrank* [26] of single-server index codes to define the optimal length of linear embedded index codes. Further, the authors also present heuristic constructions for general and specialized linear codes which have some nice properties.

As the embedded index coding problem clearly has a direct mapping with the data exchange problem considered in the present work, we can apply our data exchange bound directly to obtain a new lower bound for the communication cost of embedded index coding. The expression of this bound would be in the same form (up to only the change in notation) as Theorem 1 itself. As our bound holds in the information-theoretic sense, it would apply to not just the linear codes considered in [12] but nonlinear embedded index codes as well.

## 7. Conclusions

We have presented an information theoretic converse result for a generic data exchange problem, where the terminals contain some data in their local storage and want other data available at the local storage of other nodes. As a number of recently studied multi-terminal communication problems fall under this setting, we have used our general converse to obtain converses in many such settings, thus recovering many existing results and presenting some new results as well. Using a connection with index coding, we also presented some ideas on why and when our data exchange based converse can be loose in the index coding setting. It would be quite interesting to see if our converse result can be tightened further while still retaining a closed form expression, so as to cover all known bounds for any existing setting that can be modeled in the data exchange framework. A lower bound for the communication load in a generic data exchange setting in the presence of coded storage bits would also be a prospective direction for future research in this area.

## Data Availability

Not applicable.

## Appendix A. Proof of Theorem 1

We assume that all the bits in the collection *B* as in Definition 1 are i.i.d uniformly distributed on {0,1}. For a given communication scheme for the given data exchange problem, let $X_{i}≜\phi_{i}\left(C_{i}\right)$ represent the codeword transmitted by node *i*. For a subset S⊂[K], let $X_{S}≜\cup_{i\in S}X_{i}$. Furthermore, let $Y_{S}={⋃}_{i\in S}\left(D_{i}\cup C_{i}\right)$. We first prove the following claim.

**Claim** **A1.**
*For any S⊂[K],*

(A1) $$\begin{array}{c} H\left(X_{S}|Y_{\bar{S}}\right)\geq \sum_{P\subset S}\sum_{Q\subset S\setminus P}\frac{\left|P\right|}{|P|+|Q|-1}a_{P}^{Q}, \end{array}$$

*where $\bar{S}=\left[K\right]\setminus S$.*

Applying S=[K] to the above claim then gives Theorem 1, as $L^{*}\geq H\left(X_{\left[K\right]}\right).$

Now, we prove Claim A1. For this, we use induction on |S|.

We take the base case to be |S|=2, as for |S|=1 the problem of data exchange is not well defined. Let S={1,2} without loss of generality. Then, the LHS of (A1) gives

$$\begin{array}{cc} H(X_{1},X_{2}|Y_{\bar{S}}) & \geq H\left(X_{1}|Y_{\bar{S}}\right)+H\left(X_{2}|Y_{\bar{S}},X_{1}\right) \end{array}$$

(A2) $$\begin{array}{cc} \; & \geq H\left(X_{1}|Y_{\bar{S}},C_{2}\right)+H\left(X_{2}|Y_{\bar{S}},C_{1}\right) \end{array}$$

(A3) $$\begin{array}{cc} \; & =H\left(X_{1},D_{2}|Y_{\bar{S}},C_{2}\right)+H\left(X_{2},D_{1}|Y_{\bar{S}},C_{1}\right) \end{array}$$

(A4) $$\begin{array}{cc} \; & \geq H\left(D_{2}|Y_{\bar{S}},C_{2}\right)+H\left(D_{1}|Y_{\bar{S}},C_{1}\right) \end{array}$$

(A5) $$\begin{array}{cc} \; & \geq a_{\left\{2\right\}}^{\left\{1\right\}}+a_{\left\{1\right\}}^{\left\{2\right\}}, \end{array}$$

where (A2) follows as conditioning reduces entropy and $H(X_{1}|C_{1})=0$, (A3) is true as $H(D_{2}|Y_{\bar{S}},C_{2},X_{1})=0$ and $H(D_{1}|Y_{\bar{S}},C_{1},X_{2})=0$. This proves the base case.

We now assume that the statement is true for |S|=t−1, and prove that it holds for |S|=t. We have the LHS of (A1) satisfying the following relationships for |S|=t.

$$\begin{array}{cc} H(X_{S}|Y_{\bar{S}}) & =\frac{1}{t}\sum_{k\in S}\left(H\left(X_{S\setminus k}|Y_{\bar{S}},X_{k}\right)+H\left(X_{k}|Y_{\bar{S}}\right)\right) \end{array}$$

(A6) $$\begin{array}{cc} \; & \geq \frac{1}{t}\left(\sum_{k\in S}H\left(X_{S\setminus k}|Y_{\bar{S}},C_{k}\right)\right)+\frac{1}{t}H\left(X_{S}|Y_{\bar{S}}\right)\\ \; & \geq \frac{1}{t-1}\sum_{k\in S}H\left(X_{S\setminus k}|Y_{\bar{S}},C_{k}\right) \end{array}$$

(A7) $$\begin{array}{cc} \; & =\frac{1}{t-1}\sum_{k\in S}H\left(X_{S\setminus k},D_{k}|Y_{\bar{S}},C_{k}\right) \end{array}$$

(A8) $$\begin{array}{cc} \; & =\frac{1}{t-1}\sum_{k\in S}\left(H\left(D_{k}|C_{k},Y_{\bar{S}}\right)+H\left(X_{S\setminus k}|Y_{\bar{S\setminus k}}\right)\right), \end{array}$$

where (A6) follows because $H(X_{k}|C_{k})=0$. In (A7), we introduce $D_{k}$ freely, because

$$\begin{array}{cc} H(D_{k}|X_{S\setminus k},Y_{\bar{S}},C_{k}) & \leq H(D_{k}|X_{S\setminus k},C_{\bar{S}},C_{k})\\ \; & \leq H(D_{k}|X_{S\setminus k},X_{\bar{S}},C_{k})\\ \; & =0, \end{array}$$

where the last two statements follow because $H(X_{\bar{S}}|C_{\bar{S}})=0$ and from the decoding condition, respectively. We now interpret the two terms of (A8). For the first term, we have

(A9) $$\begin{array}{cc} \sum_{k\in S}H\left(D_{k}|C_{k},Y_{\bar{S}}\right)= & \sum_{k\in S}\sum_{P^{\prime }\subset S\setminus k}\sum_{Q\subset S\setminus (P^{\prime }\cup k)}a_{k\cup P^{\prime }}^{Q}\\ \; & =\sum_{P\subset S}\sum_{Q\subset S\setminus P}\left|P\right|a_{P}^{Q}, \end{array}$$

where the last statement follows by noting that for a fixed choice of P,Q we have |P| choices for $\left(k,P^{\prime }\right)$ such that $P^{\prime }\cup k=P$.

Now, using the induction hypothesis for the last term of (A8),

$$\begin{array}{cc} \sum_{k\in S} & H(X_{S\setminus k}|Y_{\bar{S\setminus k}}) \end{array}$$

(A10) $$\begin{array}{cc} \; & \geq \sum_{k\in S}\sum_{P\subset S\setminus k}\sum_{Q\subset S\setminus (P\cup k)}\frac{\left|P\right|}{|P|+|Q|-1}a_{P}^{Q}, \end{array}$$

(A11) $$\begin{array}{cc} \; & =\sum_{P\subset S}\sum_{Q\subset S\setminus P}\left(t-|P|-|Q|\right)\frac{\left|P\right|}{|P|+|Q|-1}a_{P}^{Q} \end{array}$$

where the above follows by noting that for a fixed choice of disjoint subsets P,Q of *S*, we have |S|−|P|−|Q| choices for *k* such that P⊂S∖k and Q⊂S∖(P∪k).

Using (A9) and (A11) we have

$$\begin{array}{cc} \mathrm{RHS}\; & \mathrm{of}\;(A8)\\ \; & \geq \frac{1}{t-1}\sum_{P\subset S}\sum_{Q\subset S\setminus P}\left|P\right|\left(1+\frac{t-|P|-|Q|}{|P|+|Q|-1}\right)a_{P}^{Q}\\ \; & =\sum_{P\subset S}\sum_{Q\subset S\setminus P}\frac{\left|P\right|}{|P|+|Q|-1}a_{P}^{Q}, \end{array}$$

thus proving Claim A1, which also concludes the proof of the theorem.

## Appendix B. Proof of (11)

We proceed according to the three steps in Section 1.2, however with some important variations that are required to prove (11).

**Applying Theorem 1 and symmetrizing:** As in the proof of Theorem 2, we use the index 0 to represent the server. Consider that, for the given placement scheme ζ, the delivery scheme is designed so that the optimal communication load $L_{c}^{*}\left(N_{u},\zeta \right)$ is achieved. Let A=[K]Nu be the set of all $N_{u}$-sized subsets of clients. Consider the coded caching subproblem induced by the server and a set A∈A of clients. Consider some demand vector $d=(d_{1},\cdots ,d_{K})$ such that the demands of the clients in *A* are distinct, i.e., $d_{i}\neq d_{j}$ for i,j∈A,i≠j.

Let Ω consist of the N−1 cyclic permutations of (1,⋯,N) which are *N*-cycles, along with the identity permutation. For σ∈Ω, let σ(d) denote the demand vector in which the ith component is exactly the value obtained by applying the permutation σ on the ith component of d, i.e., $\sigma {\left(d\right)}_{i}=\sigma \left(d_{i}\right).$

Clearly, for each σ∈Ω, we have $L_{c}^{*}\left(N_{u},\zeta \right)\geq L_{A}^{*}\left(\sigma \left(d\right)\right),$ where $L_{A}^{*}\left(\sigma \left(d\right)\right)$ is the optimal communication load for this subproblem with demands σ(d) with respect to the placement ζ.

Now, for each σ∈Ω, following similar steps as in proof of Theorem 2, we can use our data exchange bound in Theorem 1 to obtain

(A12) $$\begin{array}{c} L_{c}^{*}\left(N_{u},\zeta \right)\geq L_{A}^{*}\left(\sigma \left(d\right)\right)\geq \sum_{k\in A}\sum_{Q_{1}\subset A\setminus k}\frac{1}{1+|Q_{1}|}f_{k}^{Q_{1}}\left(\sigma \left(d\right)\right), \end{array}$$

where $f_{k}^{Q_{1}}\left(\sigma \left(d\right)\right)$ is the number of bits of the demanded file $W_{\sigma {\left(d\right)}_{k}}$ of the client *k* available at the nodes $Q_{1}\subset A$ and no other nodes in *A*. Let $a^{Q}$ be the number of all bits (corresponding to all *N* files) stored exclusively in clients Q∪{0}⊂[K]∪{0}. Then, by the structure of the demand vectors in the set D={σ(d):σ∈Ω}, we see that

$$\begin{array}{c} \sum_{\sigma \in \Omega }f_{k}^{Q_{1}}\left(\sigma \left(d\right)\right)=\sum_{Q_{2}\subset \left[K\right]\setminus A}a^{Q_{1}\cup Q_{2}}. \end{array}$$

By averaging (A12) across the *N* demand vectors in {σ(d):σ∈Ω}, we get

(A13) $$\begin{array}{c} L_{c}^{*}\left(N_{u},\zeta \right)\geq \frac{1}{N}\sum_{k\in A}\sum_{Q_{1}\subset A\setminus k}\sum_{Q_{2}\subset \left[K\right]\setminus A}\frac{1}{1+|Q_{1}|}a^{Q_{1}\cup Q_{2}}. \end{array}$$

For each A∈A, the bound (A13) holds. Averaging these bounds for all A∈A, we obtain

(A14) Lc*(Nu,ζ)≥1NKNu∑A∈A∑k∈A∑Q1⊂A∖k∑Q2⊂[K]∖A11+|Q1|aQ1∪Q2.

In the above summation, for any given A, for fixed $Q_{1}\subset A,Q_{2}\subset \left[K\right]\setminus A,$ the variable *k* takes values from $A\setminus Q_{1}.$ Thus, we have

(A15) Lc*(Nu,ζ)≥1NKNu∑A∈A∑Q1⊂A∑Q2⊂[K]∖ANu−|Q1|1+|Q1|aQ1∪Q2

(A16) =1NKNu∑q=0Nu∑Q⊂[K]∑A∈A:|Q∩A|=qNu−q1+qaQ.

For some Q⊂[K], to obtain an A∈A such that |A∩Q|=q, we have to choose *q* elements from *Q* and $N_{u}-q$ elements from outside *Q*. Thus, we have |{A∈A:|Q∩A|=q}|=|Q|qK−|Q|Nu−q. Thus, we have

(A17) Lc*(Nu,ζ)≥1NKNu∑q=0Nu∑Q⊂[K]|Q|qK−|Q|Nu−qNu−q1+qaQ.

Now

(A18) |Q|qK−|Q|Nu−qNu−q1+q=|Q|!(K−|Q|)!(Nu−q)q!(|Q|−q)!(Nu−q)!(K−|Q|−Nu+q)!(1+q)

(A19) =KNuNuq+1K−Nu|Q|−qK|Q|.

Further, we have

(A20) ∑q=0NuNuq+1K−Nu|Q|−q=∑q=1NuNuqK−Nu|Q|−q+1=K|Q|+1−K−Nu|Q|+1.

Using (A20) and (A19) in (A17), we get

(A21) Lc*(Nu,ζ)≥1N∑Q⊂[K]K|Q|+1−K−Nu|Q|+1K|Q|aQ≥1NF∑Q⊂[K]gNu(|Q|)aQ,

where $g_{{}_{N_{u}}}$ is the lower convex envelope as defined in Remark 2.

**Using the constraints to revise (A21)**:

Observe that $\sum_{Q\subset [K]}a^{Q}=NF$. We assume without loss of generality that all caches are completely populated with the bits of the file library (as we are bounding the optimal load), and thus we have $\sum_{Q\subset [K]}\left|Q\right|a^{Q}=tNF.$ By using these and applying Jensen’s inequality to (A21), we have (11). This completes the proof.

## Appendix C. Proof of (21)

The proof uses the same approach as in Appendix B, but takes into account the fact that there is no central server and the cache sizes are heterogeneous. The choice of the set of demand vectors used for symmetrization is the same as in Appendix B.

**Applying Theorem 1 and symmetrizing:** Let the sets A and A∈A, the set of cyclic permutations Ω, demand vector d, and optimal communication loads $L_{c}^{*}\left(N_{u},\zeta \right)$ and $L_{A}^{*}\left(\sigma \left(d\right)\right)$ be defined as in Appendix B. Throughout this proof we will assume $N_{u}=\min\left\{N,K\right\}$. Applying our main result Theorem 1 to the subproblem induced by the demands of the subset of nodes *A*, we obtain

$$\begin{array}{c} L_{c}^{*}\left(N_{u},\zeta \right)\geq L_{A}^{*}\left(\sigma \left(d\right)\right)\geq \sum_{k\in A}\sum_{Q\subset [K]\setminus k}\frac{1}{\left|Q\right|}c_{k}^{Q}\left(\sigma \left(d\right)\right), \end{array}$$

where $c_{k}^{Q}\left(\sigma \left(d\right)\right)$ is the number of bits of the file $W_{{\left(\sigma \left(d\right)\right)}_{k}}$ demanded by node *k* available exclusively in all nodes in *Q* and not available in [K]∖Q. Let $a^{Q}$ be the total number of bits stored exclusively in the nodes *Q*. Then, considering the set of *N* demands {σ(d):σ∈Ω}, we have

$$\begin{array}{c} \sum_{\sigma \in \Omega }c_{k}^{Q}\left(\sigma \left(d\right)\right)=a^{Q}. \end{array}$$

Averaging over all possible σ, we obtain

$$\begin{array}{c} L_{c}^{*}\left(N_{u},\zeta \right)\geq \frac{1}{N}\sum_{\sigma \in \Omega }L_{A}^{*}\left(\sigma \left(d\right)\right)\geq \frac{1}{N}\sum_{k\in A}\sum_{Q\subset [K]\setminus k}\frac{1}{\left|Q\right|}a^{Q}. \end{array}$$

Again, averaging this inequality over all possible choices of A∈A=[K]Nu, we obtain

(A22) Lc*(Nu,ζ)≥1NKNu∑A∈A∑σ∈ΩLA*(σ(d))≥1NKNu∑A∈A∑k∈A∑Q⊂[K]∖k1|Q|aQ.

In (A22), for a given choice of Q⊂[K], the term $\frac{1}{\left|Q\right|}a^{Q}$ appears in the summation for every choice of (A,k) such that k∉Q, $\left|A\right|=N_{u}$, k∈A. Therefore, the number of times $\frac{1}{\left|Q\right|}a^{Q}$ appears in (A22) is the number of such choices of (A,k) which is (K−|Q|)K−1Nu−1. Hence, we arrive at

(A23) Lc*(Nu,ζ)≥1NKNu∑Q⊂[K]K−1Nu−1(K−|Q|)|Q|aQ=FNuK∑Q⊂[K](K−|Q|)|Q|aQNF.

**Using the constraints to revise (A23)**: We use the observations that $\frac{K-x}{x}$ is convex and decreasing in x>0, $\sum_{Q\subset [K]}\frac{a^{Q}}{NF}=1$, and $\sum_{Q\subset [K]}\left|Q\right|a^{Q}\leq \sum_{i=1}^{t}K_{T_{i}}\gamma_{T_{i}}NF$ since this is the total available cache memory across all nodes. Applying Jensen’s inequality on (A23) using these constraints, we obtain

$$\begin{array}{c} L_{c}^{*}\left(N_{u},\zeta \right)\geq \frac{FN_{u}}{K}\frac{K-\sum_{i=1}^{t}K_{T_{i}}\gamma_{T_{i}}}{\sum_{i=1}^{t}K_{T_{i}}\gamma_{T_{i}}}. \end{array}$$

This completes the proof.

## Appendix D. Proof of Proposition 3

We will continue to use the notations used in the proof of Theorem 1. Let k∈[K], and $S=\{\gamma_{k},\cdots ,\gamma_{K}\}$. Note that $\bar{S}=\left\{\gamma_{1},\cdots ,\gamma_{k-1}\right\}$. We will prove by induction on |S|=K−k+1 that

(A24) $$H\left(X_{0}|Y_{\bar{S}}\right)\geq \sum_{i=k}^{K}\;\sum_{\begin{array}{c} P\subset \{\gamma_{i},\cdots ,\gamma_{K}\}\\ \gamma_{i}\in P \end{array}}\;\sum_{Q\subset \{\gamma_{i+1},\cdots ,\gamma_{K}\}}c_{P}^{Q}.$$

Then, the result claimed in the proposition follows by using $S=\left\{\gamma_{1},\cdots ,\gamma_{K}\right\}=\left[K\right]$, i.e., k=1. When |S|=1, i.e., k=K and $S=\{\gamma_{K}\}$, clearly (A24) is true, as

$$H\left(X_{0}|Y_{\left\{\gamma_{1},\cdots ,\gamma_{K-1}\right\}}\right)\geq a_{\left\{\gamma_{K}\right\}}^{\left\{0\right\}}=c_{S}^{\emptyset }.$$

Now, consider $S=\{\gamma_{k},\cdots ,\gamma_{K}\}$. The induction hypothesis is

(A25) $$H\left(X_{0}|Y_{\bar{S\setminus \gamma_{k}}}\right)\geq \sum_{i=k+1}^{K}\;\sum_{\begin{array}{c} P\subset \{\gamma_{i},\cdots ,\gamma_{K}\}\\ \gamma_{i}\in P \end{array}}\;\sum_{Q\subset \{\gamma_{i+1},\cdots ,\gamma_{K}\}}c_{P}^{Q}.$$

Using the fact $H\left(D_{\gamma_{k}}|Y_{\bar{S}},C_{\gamma_{k}},X_{0}\right)\leq H\left(D_{\gamma_{k}}|C_{\gamma_{k}},X_{0}\right)=0$, we have

(A26) $$\begin{array}{cc} H(X_{0}|Y_{\bar{S}}) & \geq H(X_{0}|Y_{\bar{S}},C_{\gamma_{k}})\\ \; & =H\left(X_{0}|Y_{\bar{S}},C_{\gamma_{k}}\right)+H\left(D_{\gamma_{k}}|Y_{\bar{S}},C_{\gamma_{k}},X_{0}\right)\\ \; & =H(X_{0},D_{\gamma_{k}}|Y_{\bar{S}},C_{\gamma_{k}})\\ \; & =H\left(D_{\gamma_{k}}|Y_{\bar{S}},C_{\gamma_{k}}\right)+H\left(X_{0}|C_{\gamma_{k}},D_{\gamma_{k}},Y_{\bar{S}}\right)\\ \; & =\sum_{\begin{array}{c} P\subset \{\gamma_{k},\cdots ,\gamma_{K}\}\\ \gamma_{k}\in P \end{array}}\;\sum_{Q\subset \{\gamma_{k+1},\cdots ,\gamma_{K}\}}\;\;\;\;\;\;\;\;c_{P}^{Q}+H\left(X_{0}|Y_{\bar{S\setminus \gamma_{k}}}\right) \end{array}$$

We observe that (A24) follows from (A25) and (A26).

## Appendix E. Proof of Theorem 5

We prove this theorem by showing that α is both upper and lower bounded by the right hand side of (43).

*Upper Bound:* Assume that H is a largest generalized independent set. We will now identify a permutation $\pi =(\pi_{1},\cdots ,\pi_{K})$ corresponding to H. Let $I_{1}=H$, and observe that as $I_{1}$ is itself a subset of H, it must contain an information bit, say $w_{P,m}^{Q}$ that is demanded by a node, say $\pi_{1}$, and none of the bits in $I_{1}\setminus \left\{w_{P,m}^{Q}\right\}$ is available as side information at $\pi_{1}$. For k=2,⋯,K, we sequentially identify $\pi_{k}$ as follows. We first define

$$I_{k}=H\;\;\setminus \;\;\underset{i<k}{⋃}\underset{P:\pi_{i}\in P}{⋃}\underset{Q\subset [K]\setminus P}{⋃}\left\{w_{P,m}^{Q}\;:\;m=1,\cdots ,c_{P}^{Q}\right\},$$

which is H minus all the bits demanded by any of $\pi_{1},\cdots ,\pi_{k-1}$. Thus, any bit in $I_{k}$ is demanded by one or more of the nodes in $\left[K\right]\setminus \left\{\pi_{1},\cdots ,\pi_{k-1}\right\}$. As $I_{k}\subset H$, it contains an information bit such that this bit is demanded by a node, say $\pi_{k}\in \left[K\right]\setminus \left\{\pi_{1},\cdots ,\pi_{k-1}\right\}$, and the rest of $I_{k}$ is not available as side information at $\pi_{k}$.

Observe that $H=I_{1}⊃I_{2}⊃\cdots ⊃I_{K}$, and $I_{k}\setminus I_{k+1}$ is the set of bits in H that are demanded by $\pi_{k}$ but not by any of the nodes in $\pi_{1},\cdots ,\pi_{k-1}$. Thus,

$$I_{1}\setminus I_{2},\;I_{2}\setminus I_{3},\cdots ,\;I_{K-1}\setminus I_{K},\;I_{K}$$

form a partition of H. Here, we have abused the notation to denote $I_{K}$ by $I_{K}\setminus I_{K+1}$. We also observe that for any choice of $k^{\prime }$ none of the bits of $I_{k^{\prime }}$ is available as side information at $\pi_{k^{\prime }}$. If $k>k^{\prime }$, as $I_{k}\subset I_{k^{\prime }}$, we deduce that none of the bits in $I_{k}$ is available as side information at $\pi_{k^{\prime }}$. Thus, we conclude that each bit in $I_{k}\setminus I_{k+1}$ is demanded by $\pi_{k}$ and is neither demanded by and nor available as side information at any of $\pi_{1},\cdots ,\pi_{k-1}$. Therefore, $|I_{k}\setminus I_{k+1}|$ is upper bounded by the number of bits exclusively demanded by $\pi_{k}$ and possibly some subset of $\left\{\pi_{k+1},\cdots ,\pi_{K}\right\}$ and which are also exclusively available at some subset of $\left\{\pi_{k+1},\cdots ,\pi_{K}\right\}$, i.e.,

$$|I_{k}\setminus I_{k+1}|\leq \sum_{\begin{array}{c} P\subset \{\pi_{k},\cdots ,\pi_{K}\}\\ \pi_{k}\in P \end{array}}\;\sum_{Q\subset \{\pi_{k+1},\cdots ,\pi_{K}\}}c_{P}^{Q}.$$

This provides us the following upper bound,

$$\begin{array}{cc} \alpha & =\left|H\right|=\sum_{k=1}^{K}\left|I_{k}\setminus I_{k+1}\right|\\ \; & \leq \sum_{k=1}^{K}\sum_{\begin{array}{c} P\subset \{\pi_{k},\cdots ,\pi_{K}\}\\ \pi_{k}\in P \end{array}}\;\sum_{Q\subset \{\pi_{k+1},\cdots ,\pi_{K}\}}c_{P}^{Q}\\ \; & \leq \max_{\gamma }\;\;\sum_{k=1}^{K}\sum_{\begin{array}{c} P\subset \{\gamma_{k},\cdots ,\gamma_{K}\}\\ \gamma_{k}\in P \end{array}}\;\sum_{Q\subset \{\gamma_{k+1},\cdots ,\gamma_{K}\}}c_{P}^{Q}, \end{array}$$

where the maximization is over all permutations γ of [K].

*Lower bound:* We derive the lower bound by showing that, for any permutation γ, the set

$$H=\overset{K}{\underset{k=1}{⋃}}\underset{\begin{array}{c} P\subset \{\gamma_{k},\cdots ,\gamma_{K}\}\\ \gamma_{k}\in P \end{array}}{⋃}\underset{\begin{array}{c} Q\subset \\ \left\{\gamma_{k+1},\cdots ,\gamma_{K}\right\} \end{array}}{⋃}\left\{w_{P,m}^{Q}\;:\;m=1,\cdots ,c_{P}^{Q}\right\}$$

is a generalized independent set. Then,

$$\alpha \geq \max_{\gamma }\left|H\right|=\max_{\gamma }\;\;\sum_{k=1}^{K}\sum_{\begin{array}{c} P\subset \{\gamma_{k},\cdots ,\gamma_{K}\}\\ \gamma_{k}\in P \end{array}}\;\sum_{Q\subset \{\gamma_{k+1},\cdots ,\gamma_{K}\}}c_{P}^{Q}.$$

To show that H is a generalized independent set, consider any subset I⊂H. Let *k* be the smallest integer such that I contains an information bit $w_{P,m}^{Q}$ with $\gamma_{k}\in P$, i.e., *k* is the smallest integer such that $\gamma_{k}$ demands some information bit in I. Therefore, any other bit $w_{P^{\prime },m^{\prime }}^{Q^{\prime }}$ in I must satisfy

$$P^{\prime }\subset \left\{\gamma_{k^{\prime }},\cdots ,\gamma_{K}\right\},Q^{\prime }\subset \left\{\gamma_{k^{\prime }+1},\cdots ,\gamma_{K}\right\}\;\mathrm{for}\;\mathrm{some}\;k^{\prime }\geq k.$$

Clearly, this bit is not available as side information at $\gamma_{k}$. Thus, H is a generalized independent set.

## Appendix F. Proof of Theorem 6

For unicast problems $c_{P}^{Q}>0$ only if |P|=1. We abuse the notation mildly and use $c_{k}^{Q}$ to denote $c_{\left\{k\right\}}^{Q}$.

*The Necessity Part:* The lower bound of Proposition 3 for unicast problems is $\sum_{i=1}^{K}\sum_{Q\subset \{\gamma_{i+1},\cdots ,\gamma_{K}\}}c_{\gamma_{i}}^{Q}$. For brevity, we will denote this sum as f(γ). For Theorem 4 to be tight it is necessary that the bound of this theorem be equal to α, i.e., the value of *f* be the same for all permutations γ.

We first show that $c_{i}^{\left\{j\right\}}=c_{j}^{\left\{i\right\}}$ for any i≠j. Consider two permutations γ and π which differ only in the two coordinates K−1,K, given as, $\gamma_{K-1}=i,\gamma_{K}=j$ and $\pi_{K-1}=j,\pi_{K}=i$. Then,

$$\begin{array}{cc} 0 & =f\left(\gamma \right)-f\left(\pi \right)=c_{i}^{\left\{j\right\}}+c_{i}^{\emptyset }+c_{j}^{\emptyset }-c_{j}^{\left\{i\right\}}-c_{j}^{\emptyset }-c_{i}^{\emptyset }\\ \; & =c_{i}^{\left\{j\right\}}-c_{j}^{\left\{i\right\}}. \end{array}$$

This proves the result for |S|=2. Next, we will assume that the necessity part is true for any S⊂[K] of size less than or equal to *t*, and use induction to prove the result for |S|=t+1.

Given any (t+1)-set S⊂[K] and any $k,k^{\prime }\in S$ we will now show that $c_{k}^{S\setminus k}=c_{k^{\prime }}^{S\setminus k^{\prime }}$. Consider two permutations γ,π that differ only in the two coordinates K−t,K−t+1, and

$$\begin{array}{cc} \; & \;\;\gamma_{K-t},\cdots ,\gamma_{K},\pi_{K-t},\cdots ,\pi_{K}\in S,\\ \; & \gamma_{K-t}=k,\gamma_{K-t+1}=k^{\prime },\;\;\mathrm{and}\;\;\pi_{K-t}=k^{\prime },\pi_{K-t+1}=k. \end{array}$$

We observe that $S=\left\{\gamma_{K-t},\cdots ,\gamma_{K}\right\}=\left\{\pi_{K-t},\cdots ,\pi_{K}\right\},$ and

(A27) $$\begin{array}{cc} 0 & =f(\gamma )-f(\pi )\\ \; & =\sum_{Q\subset \{\gamma_{K-t+1,\cdots ,\gamma_{K}}\}}\;\;\;\;\;\;\;c_{k}^{Q}\;\;\;+\;\;\;\;\sum_{Q\subset \{\gamma_{K-t+2,\cdots ,\gamma_{K}}\}}\;\;\;\;\;\;\;c_{k^{\prime }}^{Q}-\;\;\;\;\;\;\sum_{Q\subset \{\pi_{K-t+1,\cdots ,\pi_{K}}\}}\;\;\;\;\;\;\;c_{k^{\prime }}^{Q}\;\;\;-\;\;\;\;\sum_{Q\subset \{\pi_{K-t+2,\cdots ,\pi_{K}}\}}\;\;\;\;\;\;\;c_{k}^{Q}\\ \; & =\sum_{Q\subset S\setminus k}c_{k}^{Q}+\sum_{Q\subset S\setminus \{k,k^{\prime }\}}\;\;\;c_{k^{\prime }}^{Q}-\sum_{Q\subset S\setminus k^{\prime }}c_{k^{\prime }}^{Q}-\sum_{Q\subset S\setminus \{k,k^{\prime }\}}\;\;\;c_{k}^{Q}. \end{array}$$

We now argue that except for the two terms $c_{k}^{S\setminus k}$ and $-c_{k^{\prime }}^{S\setminus k^{\prime }}$ all other terms in (A27) cancel out. Consider any term in the first summation of (A27) with |Q|≤t−1. If $k^{\prime }\in Q$, then by the induction hypothesis, the term $-c_{k^{\prime }}^{Q\cup k\setminus k^{\prime }}$ present in the third summation will cancel $c_{k}^{Q}$. If $k^{\prime }\notin Q$, then $k,k^{\prime }\notin Q$, and the term $-c_{k}^{Q}$ in the fourth summation will cancel $c_{k}^{Q}$. Similarly, every term $c_{k^{\prime }}^{Q}$ in the second summation will cancel the corresponding term $-c_{k^{\prime }}^{Q}$ in the third summation. It is straightforward to observe that these correspondences between the positive and negative terms are unique, and thus we are left with $0=c_{k}^{S\setminus k}-c_{k^{\prime }}^{S\setminus k^{\prime }}$.

*The Sufficiency Part:* The lower bound in Theorem 4 is

$$\begin{array}{c} L^{*}\geq \sum_{k=1}^{K}\sum_{Q\subset [K]\setminus k}\frac{1}{1+|Q|}c_{k}^{Q}=\sum_{k=1}^{K}c_{k}^{\emptyset }+\sum_{\begin{array}{c} S\subset [K]\\ |S|\geq 2 \end{array}}\sum_{k\in S}\frac{c_{k}^{S\setminus k}}{\left|S\right|}. \end{array}$$

This lower bound can be met by a scheme that uses a combination of uncoded transmission and clique covering. All the bits that are not available at any of the *K* clients are transmitted uncoded incurring the cost $\sum_{k=1}^{K}c_{k}^{\emptyset }$. For every S⊂[K] with |S|≥2, the encoder constructs |S| vectors, one corresponding to each k∈S and broadcasts the XOR of these vectors to the clients. The vector for k∈S consists of the $c_{k}^{S\setminus k}$ bits demanded by node *k* and available at nodes S∖k. All these |S| vectors have the same length $c_{k}^{S\setminus k}$. These coded transmissions incur an additional cost $\sum_{\begin{array}{c} S\subset [K]\\ |S|\geq 2 \end{array}}\sum_{k\in S}\frac{c_{k}^{S\setminus k}}{\left|S\right|}$, thereby achieving the lower bound. This is the well known clique-covering index coding scheme (see [27,28]) and these transmissions allow the clients to decode their demands.
